# WHO mental health gap action programme (mhGAP) intervention guide: updated systematic review on evidence and impact

**DOI:** 10.1136/ebmental-2021-300254

**Published:** 2021-04-26

**Authors:** Roxanne Keynejad, Jessica Spagnolo, Graham Thornicroft

**Affiliations:** 1 Department of Health Service and Population Research, Institute of Psychiatry, Psychology and Neuroscience, King's College London, London, UK; 2 Département des Sciences de la Santé Communautaire, Université de Sherbrooke, Sherbrooke, Quebec, Canada; 3 Centre de recherche Charles-Le Moyne – Saguenay–Lac-Saint-Jean sur les innovations en santé, Campus de Longueuil, Université de Sherbrooke, Sherbrooke, Quebec, Canada

**Keywords:** adult psychiatry, child & adolescent psychiatry, depression & mood disorders, schizophrenia & psychotic disorders, substance misuse

## Abstract

**Question:**

There is a large worldwide gap between the service need and provision for mental, neurological and substance use disorders. WHO’s Mental Health Gap Action Programme (mhGAP) intervention guide (IG), provides evidence-based guidance and tools for assessment and integrated management of priority disorders. Our 2017 systematic review identified 33 peer-reviewed studies describing mhGAP-IG implementation in low-income and middle-income countries.

**Study selection and analysis:**

We searched MEDLINE, Embase, PsycINFO, Web of Knowledge, Scopus, CINAHL, LILACS, ScieELO, Cochrane, PubMed databases, 3ie, Google Scholar and citations of our review, on 24 November 2020. We sought evidence, experience and evaluations of the mhGAP-IG, app or mhGAP Humanitarian IG, from any country, in any language. We extracted data from included papers, but heterogeneity prevented meta-analysis.

**Findings:**

Of 2621 results, 162 new papers reported applications of the mhGAP-IG. They described mhGAP training courses (59 references), clinical applications (n=49), research uses (n=27), contextual adaptations (n=13), economic studies (n=7) and other educational applications (n=7). Most were conducted in the African region (40%) and South-East Asia (25%). Studies demonstrated improved knowledge, attitudes and confidence post-training and improved symptoms and engagement with care, post-implementation. Research studies compared mhGAP-IG-enhanced usual care with task-shared psychological interventions and adaptation studies optimised mhGAP-IG implementation for different contexts. Economic studies calculated human resource requirements of scaling up mhGAP-IG implementation and other educational studies explored its potential for repurposing.

**Conclusions:**

The diverse, expanding global mhGAP-IG literature demonstrates substantial impact on training, patient care, research and practice. Priorities for future research should be less-studied regions, severe mental illness and contextual adaptation of brief psychological interventions.

## Background

There is a well-reported, very sizeable global gap between the need and provision of services to prevent, identify and treat mental, neurological and substance use (MNS) disorders. To expedite care as efficiently as possible, the WHO’s World Mental Health Report^1^ recommended the assessment and management of MNS disorders in primary healthcare (PHC) and community settings, as outlined in the Mental Health Action Plan.^2^ To deliver community mental healthcare, PHC staff require training in the assessment, diagnosis and management of MNS disorders, alongside other key components, including regular supervision and support. Central to this agenda is the WHO Mental Health Gap Action Programme^3^ (mhGAP) and intervention guide (IG).^4^

The mhGAP-IG is an evidence-based tool for the assessment and treatment of priority MNS disorders, featuring guidelines for clinical decision making. Aimed at non-specialist PHC staff in low-income and middle-income countries (LMICs), the mhGAP-IG is also used by governments, non-governmental organisations and researchers. An mhGAP Humanitarian Intervention Guide (HIG),^5^ for settings characterised by widespread psychological trauma and even less access to mental health specialists, has also been published,^5^ alongside an mhGAP-IG mobile device app.^6^

Our previous systematic review^7^ identified 33 published peer-reviewed studies and protocols which used the mhGAP-IG. Given that another review found only six published studies of experimental non-communicable disease clinical guideline implementation in LMICs,^8^ the mhGAP-IG literature was relatively substantial in 2017. The mhGAP-IG had been adopted by clinicians, government ministries, trainers, educators and academics in diverse LMICs. Applications ranged from local adaptation, training and clinical practice, to mobile applications for isolated PHC staff, tablet-based avatar-assisted family training, economic modelling, novel rating scales and comparison interventions in randomised controlled trials (RCTs). The literature was, however, dominated by studies from a subset of countries, suggesting that much implementation was not evaluated, or that evaluations were not widely disseminated. Reliance on relatively limited pre-mhGAP and post-mhGAP training knowledge assessments meant that opportunities may have been missed at times, to describe real-world contextual challenges to widespread uptake and scale-up.

## Objective

In this study, we sought to identify evidence generated since the publication of our previous review for the practical implementation of the WHO mhGAP-IG and associated tools, in terms of how they have been used, evaluated and reported.

## Study selection and analysis

We included any type of study design, review or report of evidence, experience or evaluation of using the mhGAP-IG in LMICs, from any country, in any language. In order to identify as many potentially relevant records as possible, we searched the following databases on 24 November 2020: 3ie, Cochrane Library, CINAHL, EMBASE, LILACS, Medline, PsycINFO, PubMed, SciELO, Scopus, Web of Knowledge. The only search terms used were ‘mental health gap action programme’ OR ‘mental health gap action program’ OR ‘mhGAP’. The term ‘intervention guide’ was not included, due to its variable use in the literature and in practice. Searches were conducted in English, but studies written in other languages were eligible for inclusion. In addition to database searches, the reference lists of relevant excluded papers were searched for eligible studies. Grey literature, including book chapters and conference presentations, were identified by repeating the search on Google Scholar. We used forward citation tracking of our 2017 systematic review to identify additional records.^7^ This work is registered on the International Prospective Register of Systematic Reviews (registration number: CRD42017068459).

## Findings

**Figure 1:**
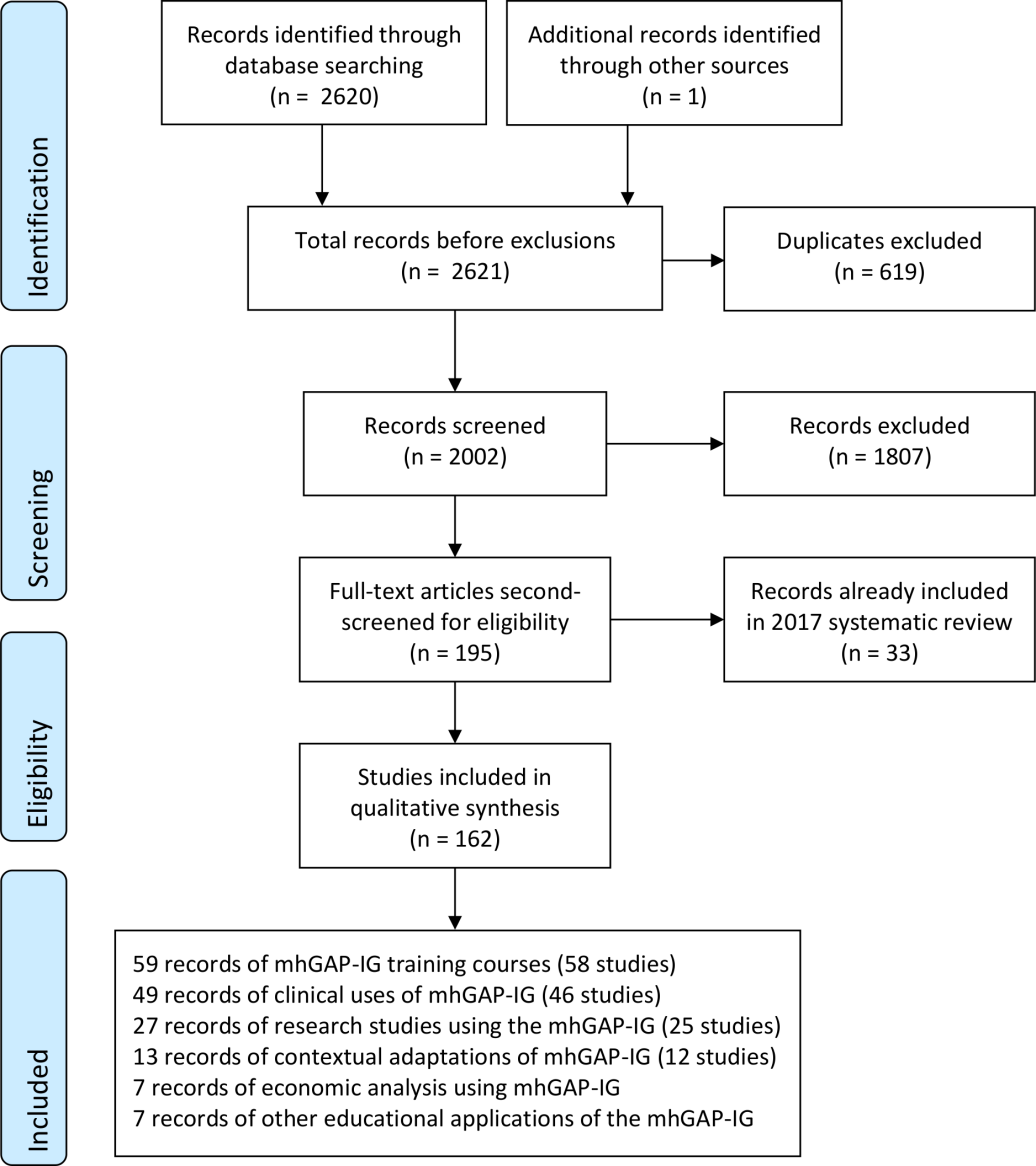
PRISMA flow diagram. mhGAP-IG, Mental Health Gap Action Programme Intervention Guide; PRISMA, Preferred Reporting Items for Systematic Reviews and Meta-Analyses.

Figure 1 shows the flow of studies from identification to screening, eligibility and inclusion. The titles and abstracts of the 2002 non-duplicated papers were screened by RK excluding 1807, which did not review or report evidence, experience or evaluation the mhGAP-IG, app or HIG. The details of a subset of excluded studies reporting non-mhGAP-IG-related integrations of mental healthcare into PHC were recorded for a separate review. The remaining 195 full-text articles were screened again for eligibility by JS and categorised into study types. Where studies could be categorised into more than one type (e.g., both clinical application and research), a judgement was made about the study’s primary focus. No papers were excluded based on language, and no relevant papers from high-income settings were identified.

Table 1 shows the distribution of study types across geographical regions. Most studies (40%, n=62) were conducted in the African region and South-East Asia (25%, n=38). Notably few studies were conducted in the Eastern Mediterranean (7%, n=10), region of the Americas (4%, n=6) and the European region (1%, n=2). Ten per cent of studies (n=15) reported results from at least two countries on different continents. Most included studies described uses of the mhGAP-IG, app or HIG for training (38%, n=58), clinical use (30%, n=44) and research (16%, n=25).

**Table 1:** Studies reporting mhGAP-IG use, experience or evaluation, by type and geographical region

Types →	Training courses	Clinical uses	Research	Contextual adaptation	Economic analysis	Other educational	Total studies
Regions ↓							
African Region	23	25	8	4	2	0	62
South-East Asia	10	13	9	3	0	3	38
Multicontinental	4	4	1	3	3	0	15
Western Pacific	7	1	2	0	1	1	12
Region of the Americas	5	1	0	0	0	0	6
Eastern Mediterranean	8	2	5	1	1	0	10
European Region	1	0	0	0	0	1	2
Other	0	0	0	1	0	2	3
Total	58	46	25	12	7	7	155

mhGAP-IG, Mental Health Gap Action Programme Intervention Guide.

Table 2 shows the study design methods used across different study types. RCTs comprised 15% (n=23) of studies and RCT protocols or trial registrations, 12% (n=19). Uncontrolled studies comprised 23% of studies (n=36), descriptive accounts 18% of studies (n=28), qualitative studies 8% (n=13) and other non-randomised designs 7% (n=10).

**Table 2:** Methods used in each category of study design

Types →	Training courses	Clinical uses	Research	Contextual adaptation	Economic analysis	Other educational	Total studies
Methods ↓							
RCT	4	8	10	0	0	1	23
RCT protocol/trial registration	3	10	6	0	0	0	19
Uncontrolled study	22	12	0	0	1	1	36
Descriptive account	16	6	1	5	0	0	28
Qualitative study	5	1	0	6	0	1	13
Other non-randomised design	4	4	1	0	1	0	10
Other protocol	1	3	3	0	0	0	7
Feasibility study	3	2	2	0	0	0	7
Economic study	0	0	0	0	5	0	5
Non-clinical study	0	0	2	1	0	4	5
Total	58	46	25	12	7	7	155

RCT, randomised controlled trial.

Of the 193 eligible studies, data were extracted by JS and RK from the 162 papers (describing 155 studies) not included in the 2017 systematic review (n=33). The heterogeneity of mhGAP-IG, app and HIG uses, outcome measures, and evaluations precluded meta-analysis. Due to the heterogeneity of designs, including a large proportion of non-randomised designs and variable reporting methods, we did not assess risk of bias across included studies.^9^ Extracted data included country involved, participants, sample, nature of use, evaluation conducted and summary of findings, where relevant. Below, we summarise the findings reported by studies categorised into six different types: use in training (online supplemental material table 4), use in clinical practice (online supplemental material table 5), use in research (online supplemental material table 6), local adaptation or contextualisation (online supplemental material table 7), economic evaluation (online supplemental material table 8) and other educational purposes (online supplemental material table 9).

10.1136/ebmental-2021-300254.supp1 Supplementary data

### Use in training

Of the 162 included papers, 58 studies (59 references) reported the use of the mhGAP-IG or HIG in training courses, trained PHC and other community-based staff (health extension workers, community health workers (CHWs), nurses, midwives, pharmacists, physician assistants, clinical officers, general practitioners, social workers), students (nursing and medical undergraduates, master’s students), research assistants, clinical educators, decision-makers, caregivers, traditional and faith healers, and mental health service users.

The impact of mhGAP-IG-based training was evaluated using cross-sectional designs, cohort studies, pretest/post-test studies, retrospective medical records reviews and RCTs. Overall, post-mhGAP-IG training, PHC and community-based staff showed increased mental health knowledge and awareness, improved attitudes towards mental ill-health and people living with mental health problems, improved attitudes towards psychiatry, more confidence in managing mental health problems in PHC, increased job satisfaction and interest in mental health training. Evaluations of mhGAP-IG-based training highlighted trainees’ satisfaction with the programme, which was generally considered useful, relevant and valuable.

Aspects of trainees’ practice also improved. Studies reported improved diagnostic skills and diagnostic agreement, increased mental health service efficiency, and greater family involvement in care. Studies also reported benefits for patients following mhGAP-IG-based staff training. These included increased mental health service utilisation, clinical improvement, returning to work, fewer discriminatory experiences and greater satisfaction with services.

Descriptive accounts and qualitative studies of mhGAP-IG-based training highlighted barriers to training PHC and community-based staff. These included limited funding or institutional support for training, difficulty establishing supervision mechanisms, cultural differences in conceptualisations of mental ill-health, the need for further training and time constraints. Descriptive accounts and qualitative studies also emphasised the importance of continued supervision, medication supplies, coordination of services, political commitment to mental healthcare, planning and leadership to the success of mhGAP-IG-based training.

Four records describing training courses were protocols or trial registrations, from Iran, Sri Lanka India and Nepal. These studies aimed to use mhGAP-IG-based training to improve youth and parent functioning, reduce mental health symptoms, improve clinical skills and reduce stigma among health workers.

### Use in clinical practice

Forty-six studies (49 references) reported clinical outcomes of applications of the mhGAP-IG or HIG in practice(online supplemental material table 5). Target patient groups included adults with mood or anxiety disorders, adults with severe mental illness (SMI), pregnant women with mood disorders, parents of primary school-aged children and people living with HIV.

Studies assessed the mhGAP-IG and HIG’s impact in clinical practice through retrospective medical records reviews, uncontrolled studies, cross-sectional studies, case–control studies and RCTs. They reported positive impacts of the mhGAP-IG on mental health awareness, clinician-confirmed case identification, mental health symptoms, clinical recovery, experiences of discrimination, food security, quality of life, contact coverage and access to care, for example, clinical consultation numbers, intervention adherence, retention in treatment and numbers of facilities treating chronic illness. Studies explored factors influencing the mhGAP-IG and HIG’s clinical uptake. These included confidential consultation space, patient trust in health workers, mental health stigma, medication supply, staff (including psychiatrist) availability, staff turnover, supervision, support and health worker motivation.

Some studies used RCT designs to evaluate clinical use of the mhGAP-IG alone, while others combined the mhGAP-IG with problem-solving therapy, behavioural activation, interpersonal therapy, parent skills training, communication skills and counselling. In some cases, the mhGAP-IG was provided to both intervention and control groups as enhanced usual care.

Ten records evaluating clinical use of the mhGAP-IG, app or HIG were protocols and three were trial registrations, from Nigeria, Ghana, Kenya, Uganda Rwanda, South Africa, Ethiopia, India, Nepal and Pakistan. These studies aimed to improve patient symptoms or functioning, parent–child communication and interaction, parent quality of life and the feasibility of combining mhGAP-IG-informed care with interpersonal therapy. One trial registration will compare the electronic mhGAP-IG with the paper version.

### Use in research

Most of the studies included in this review employed research methods in some form. We categorised 25 studies (27 references) as reporting uses of the mhGAP-IG for research where the primary purpose of the work was a research endeavour, whose conduct was facilitated by the mhGAP-IG as a tool(online supplemental material table 6). Ten studies (seven RCTs, two feasibility studies and one non-randomised trial) used the mhGAP-IG to standardise the mental healthcare received by participants in both intervention and control arms. In India, Counselling for Alcohol Problems was associated with significantly more remission and abstinence from alcohol use disorder at 3 and 12 months' follow-up than mhGAP-IG-enhanced usual care. Also in India, the Healthy Activity Programme behavioural activation intervention was associated with significantly lower depressive symptoms and increased remission at 3 and 12 months' follow-up than mhGAP-IG-enhanced usual care. In Pakistan, there was no difference between the impact of mhGAP-IG-enhanced usual care and an intensive group-based psychosocial intervention on depressive symptoms and remission at 6 and 36 months' follow-up. Also in Pakistan, there was no difference between electronic health records and decision support for integrated management of chronic conditions (including depression, using mhGAP-IG content) and non-electronic training. In Nepal, a feasibility study found that group problem management plus (PM+) was acceptable and feasible to compare with mhGAP-IG-enhanced usual care. In Somaliland, a non-randomised study found that mhGAP-IG-informed community-based relapse prevention was more effective for patients commencing the programme in remission than those requiring intensive home-based care first.

Two RCTs used the mhGAP-IG to inform the content of a novel intervention in Pakistan and China and one used it to screen for psychosis and assess suicide risk during a trial in Malaysia. In Pakistan, more pregnant women sought help for distress from CHWs following mhGAP-IG-informed psychoeducation than women in the control arm. In China, an mhGAP-IG-informed intervention was associated with greater reductions in anticipated discrimination, negative symptoms and functioning scores than a community psychiatrist interview. In Malaysia, common mental disorder symptoms improved more in participants receiving integrative adapt therapy than those receiving cognitive behavioural therapy.

One validation study used the mhGAP-IG to provide treatment for adolescents diagnosed with a mental disorder using a newly adapted screening tool in Ethiopia. The adapted Amharic Youth Self Report was a reliable and valid screen for anxiety, depression and social problems in female adolescents and attention problems in young men. One study developed a scale to categorise free Arabic-language anxiety and depression apps using the mhGAP-IG. One descriptive account incorporated the mhGAP-IG into a novel counselling aid (FELICIA) based on the Thinking Healthy Programme, for couples experiencing infertility in Nigeria; a pilot study is underway.

Nine studies using the mhGAP-IG for research were protocols: six RCTs, a feasibility study, an implementation study and a cohort study. One prospective cohort study will evaluate the impact on tuberculosis outcomes of mhGAP-IG-based depression treatment. Five RCTs will use the mhGAP-IG to standardise the mental healthcare received by participants in both intervention and control arms, in Ethiopia, Tanzania, Pakistan and Nepal. A sixth RCT protocol aims to compare the reach, costs and clinical effectiveness of mhGAP-IG-based care delivered by specialist mental health professionals, PHC staff and CHWs in Mozambique. The implementation study protocol aims to evaluate implementation strategies for cognitive stimulation therapy in Brazil, India and Tanzania.

### Local adaptation or contextualisation

Twelve studies (13 references) reported local adaptation or contextualisation of the mhGAP-IG(online supplemental material table 7). Qualitative studies, case studies and descriptive accounts included reflections and experiences of adapting or contextualising the mhGAP-IG. Methods included ethnographic research, in-depth stakeholder interviews and focus groups, theory of change workshops, literature reviews, local epidemiological evidence reviews, situational analyses and asset mapping of community resources.

Table 3 summarises facilitators and barriers to mhGAP-IG implementation identified by qualitative studies, at service user, staff, service and leadership levels. Consistent themes included integrating the biomedical model with cultural perspectives, addressing stigma, adjusting staff workloads, providing supervision, creating referral pathways and obtaining institutional and political support.

**Table 3:** Facilitators and barriers to mhGAP-IG implementation success identified by qualitative studies

System level	Facilitators	Barriers
Service user	Ability to see traditional healers alongside biomedical care.^31^ Community awareness raising.^32^	Cultural differences with the biomedical model.^33^ Different perspectives on appropriate treatment.^34–36^ Rural residence, distance from health facilities, thinking that problems will self-resolve, concerns about treatment costs.^37^ Resistance to treatment.^38^
Staff	Health worker motivation.^39^ Sharing research findings collaboratively.^40^	Resistance from faculty members.^41^ Time constraints and workload.^42 43^ Stigma.^38 39 43^ Mistrust of informal health providers, cultural misunderstandings.^38^
Service	Supervision.^39 42 44^ Onward referral.^32^ Reliable psychotropic medicine procurement.^32 39 44^ Trained female community health volunteers.^32^	Scarcity of specialist staff to deliver supervision.^39 45^ Lack of financial resources^38 41^ Limited referral systems.^38^ Staff turnover, lack of confidential space for consultation.^39^
Leadership	Strong political and organisational support.^35 36 39 40^	Lack of institutional support.^41^

Recommended considerations when adapting or contextualising the mhGAP-IG included cultural differences in explanatory models and attitudes towards mental disorders, the structure of the local health system, the availability of supervision and support post-training, trainees’ prior education, knowledge and skills, trainee recruitment processes and the wider sociopolitical context.^10^ Authors also recommended considering the availability of specialists for referral, medication supplies, translation of the IG into local languages and organisational roles.^11 12^

One study developed a framework to integrate cultural knowledge, structural competence and ethics into mhGAP-IG planning, adaptation, training and implementation. Domains included examining concepts of wellness and illness, exploring systems of care, recognising formal and informal care systems, and considering the ethical space of power dynamics and decision making.^13^

### Economic evaluation

Seven studies (seven references) used the mhGAP-IG to conduct economic evaluations(online supplemental material table 8). Three studies used the mhGAP costing tool to calculate human resource requirements from the prevalence of depression in Syria, MNS disorders in Pacific Island and 48 sub-Saharan African countries. Two studies performed cost-benefit analyses of the scale-up of effective treatments for depression and anxiety in 36 countries, and for psychosis, depression and epilepsy in Ethiopia, India, Nepal and Uganda. Two studies assessed household out-of-pocket health expenditures, finding that having a relative with SMI was associated with catastrophic health expenditure, financial dissatisfaction and cost-cutting actions, such as withdrawing children from school. Functional impairment was associated with higher treatment costs in Ethiopia and India, and out-of-pocket expenditure in Uganda and India.

### Other educational purposes

The final seven studies reported uses of the mhGAP-IG for a range of healthcare education-focused purposes(online supplemental material table 9). Two described the development of a community informant detection tool in Nepal based on mhGAP-IG modules and its cluster RCT evaluation, which found that it significantly increased diagnoses. One study developed an mhGAP-IG-based electronic decision support system in India, where it was acceptable but required modification. An Indonesian study used the mhGAP-IG to code interview responses, identifying greater mental health knowledge and lower stigma among specialist health workers, compared with non-specialists. In Ukraine, the incorporation of the mhGAP-IG into the undergraduate medical curriculum was associated with sustainability, cost-effectiveness, educational and care quality benefits. One study designed mhGAP-IG-based clinician competencies to inform training and assessment. A qualitative study used participant observations of the mhGAP-IG’s use to analyse and critique its algorithmic approach.

## Conclusions and clinical implications

This updated systematic review of the literature on the mhGAP-IG, app and HIG demonstrates a dramatic increase in published studies of its use and evaluation since 2017.^7^ Most of the literature comes from the African region and South-East Asia, with notable evidence gaps including the region of the Americas and the Eastern Mediterranean. Countries participating in the programme for improving mental healthcare (PRIME) study (Ethiopia, India, Nepal, South Africa and Uganda) are well represented, demonstrating the benefits to mental health research and evaluation of investment, shared training and methodologies. PRIME identified barriers to integrating mental health into PHC, including limited funding, insufficient specialists to supervise non-specialist workers, inadequate health system structures to support roll-out of task-shared interventions, low community awareness of mental health and high levels of stigma.^14^ Building on PRIME, the AFFIRM study is conducting two RCTs in South Africa and Ethiopia of task-shared psychological interventions, building capacity through fellowships and mentorships for early career researchers from Ethiopia, Ghana, Malawi, Uganda and Zimbabwe, and building a network for interdisciplinary collaboration.^15^ Both projects demonstrate ways of enhancing the impact and sustainability of mental health research, through long-term collaborations between LMIC and HIC institutions, funded to design and evaluate new interventions from piloting to scale-up, and develop the next generation of global mental health researchers in LMICs.

Similar study types to those identified in our 2017 review^7^ have been published since, with the highest proportions of studies reporting on mhGAP-IG training courses, clinical and research applications. The surge in literature also includes contextual adaptations, economic analyses and novel applications to health education. Studies demonstrated the benefits of the mhGAP-IG’s use in training for improving the knowledge, attitudes and confidence of health workers in relation to mental health disorders. Clinical studies identified improvements in patient symptoms, quality of life, access to and engagement with mental healthcare associated with mhGAP-IG implementation.

The published evidence comprises a breadth of research methods, with high proportions of RCTs and protocols for RCTs, and smaller proportions of related feasibility studies, implementation studies and process evaluations. The publication of uncontrolled, non-randomised, descriptive and case studies contributes a valuable literature on the mhGAP-IG from settings where more extensive research studies may not be feasible. Adaptation studies recommended a range of contextual considerations to optimise the success of mhGAP-IG implementation.

Many research uses of the mhGAP-IG aimed to adapt and evaluate brief psychological interventions for settings where specialist mental healthcare is limited or unavailable. Often, such interventions were implemented alongside mhGAP-IG training (as ‘enhanced usual care’) or as a next step after successful mhGAP-IG training for clinical use. Literature on the Thinking Healthy Programme, Healthy Activity Programme, Counselling for Alcohol Problems and PM+, largely from South Asia, demonstrates the broader role of the mhGAP-IG in encouraging the development and rigorous evaluation of acceptable, feasible and scalable talking therapies in LMICs. Sharing of intervention manuals via the WHO website should facilitate the adaptation and evaluation of these interventions for other countries, cultures and contexts. SMIs such as schizophrenia, severe depression and bipolar affective disorder in PHC were less frequently targeted by intervention studies, the majority of which focused on mild to moderate depression. Lessons learned from the use of the mhGAP-IG for common mental disorder research could now be applied to research and intervention development for people with SMI.

Recognition has grown of the need to improve the quality of inpatient and community mental healthcare, attend to the human rights of people with mental disorders and promote recovery.^16^ The fact that most mhGAP-IG, app and HIG training combined lectures and small-group tasks with role play interactions suggests that scaling up the mhGAP-IG could have wider benefits, improving health worker communication skills and the compassion and respect with which care is delivered. Twenty-one included studies addressed or measured stigma, or planned to. Of 15 completed studies, nine reported reductions in one or more measures of stigma, even where health worker knowledge or skills were unchanged.^17–25^ Economic studies identified the scale of the mental health gap, its financial^26^ and disability impacts,^27^ and calculated the workforce requirements^28–30^ and costs of treatment.

The principal limitation of this study is the likelihood of publication bias, with studies finding no significant or negative results less likely to have been published. A substantial number of reports is likely to remain in the ‘grey literature’ outside peer reviewed journals. A further limitation is that we conducted single-author screening of potentially eligible studies, raising the risk that some eligible studies could have been missed.

Since their release, the WHO mhGAP-IG, app and HIG have made a remarkable impact on global mental health education and training, clinical practice, research and policy. Included studies used a range of methods: a testament to practitioners’ commitment to mhGAP-IG monitoring, evaluation and implementation research. Promising approaches to strengthen the evidence base include consortia bringing researchers together from a range of LMICs and HICs for collaborative, interdisciplinary study, benefiting from shared learning and pooled resources. The mhGAP-IG has created a dynamic field in which practitioners from diverse international contexts learn from each other’s experiences and adapt study designs and intervention models for their own context. The next step must be to fill gaps in the evidence base for under-studied regions and disorders, and to investigate the scale-up and sustainable integration of isolated interventions into long-term clinical practice.

## Data Availability

Data sharing is not applicable as no datasets were generated and/or analysed for this study. The data analysed in this systematic review came from publications of research studies whose data may be sought by direct contact with the study authors.
