# Supplementary material for: WHO mental health gap action programme (mhGAP) intervention guide: updated systematic review on evidence and impact
Source: Evid Based Ment Health. 2021 Apr 26;24(3):124–30. doi: 10.1136/ebmental-2021-300254 (PMC8311089; doi:10.1136/ebmental-2021-300254)
Supplement: Supplementary data [file ebmental-2021-300254supp001.pdf]

Supplementary Online Material:

Table 4: Summary of included studies describing mhGAP-IG Training Courses

Table 5: Summary of included studies describing clinical uses of mhGAP-IG

Table 6: Summary of included studies reporting uses of the mhGAP-IG in Research

Table 7: Summary of included studies describing country-specific mhGAP-IG adaptation or contextualisation

Table 8: Summary of included studies using the mhGAP-IG to conduct economic analysis

Table 9: Summary of included studies reporting use of the mhGAP-IG for other educational purposes

Table 4: Summary of included studies describing mhGAP-IG Training Courses

Authors	Country	Study design	Sample	Intervention details	Evaluation details, if any	Summary of findings
**de la Barra et al. (2014) (1)**	Chile	Descriptive account	2983 out of 66753 primary care professionals and a further 3680 primary care staff.	Training was provided to 2983 staff who assist children and adolescents with mental health problems between 2016 and 2018. Training used mhGAP to teach diagnosis, detection and treatment of mental and substance use disorders in non-specialist settings. Distance training in mental health topics was delivered to 3680 additional primary care staff.	Not applicable (N/A).	N/A
**Diez-Canseco et al. (2018) (2)**	Peru	Feasibility study	PHC staff.<br><br>18+ year-old patients attending 5 participating PHC centres.	(1) Training of PHC providers using mhGAP training modules.<br><br>(2) Task shifting detection and referral of mental disorders.<br><br>(3) Mobile health (mHealth) screening app followed by motivational and reminder short message service (SMS) contacts to identify at-risk patients.	App-collected data: screening progress by PHC centres, number of patients screened (complete and incomplete), number of positive diagnoses, number of patients identified at suicide risk, actions taken by PHC staff (e.g., referral to a psychologist). Follow-up interviews with patients: motivation to seek care, perceived barriers and opinions on receiving care. Midterm postintervention interviews with PHC providers: experiences and opinions of participating, barriers and suggestions for improvement, feasibility and willingness to continue implementing screening.	733 patients were screened by the primary health care providers during routine consultations, and 762 screening were completed in total.<br><br>Chronic disease (50%, 380/762) and antenatal care services (37%, 380/762) screened the most patients.<br><br>Time constraints and workload were the main barriers to implementing screening, whereas use of technology, training, and supervision of PHC providers were facilitators.<br><br>Patients and providers recognized the utility of screening.
**World Health Organization (2020) (3)**	Mexico, Liberia, Nigeria, Ukraine	Descriptive account	Mexico: 3rd year medical students<br>Liberia: nurses, physician assistants, registered midwives<br>Nigeria: 5th year medical and MSc students<br>Ukraine: decision-makers and clinical educators.	Mexico: 20-hour mental health course included the mhGAP-IG .<br>Liberia: training attendance required pre-graduation.<br>Nigeria: 1) mhGAP-IG child and adolescent mental health, depression and dementia modules taught during 4 weeks of clerkship. 2) Child and Adolescent Mental Health MSc students received 4 weeks' mhGAP-IG training.<br>Ukraine: 1st mhGAP-IG child and adolescent pre-service training of trainers and supervisors course.	N/A	Mexico: 50 medical students trained with mhGAP-IG. They reported more knowledge about mental disorders and evaluated training positively.<br><br>Liberia: 16% of all nursing, midwifery, and physician assistant students (n=1251) completed mhGAP training.<br><br>Nigeria: >300 medical students who now practice in the community trained in youth mental health.<br><br>Ukraine: participants gained skills to enhance local teaching programmes with mhGAP-IG.

Authors	Country	Study design	Sample	Intervention details	Evaluation details, if any	Summary of findings
**Chaulagain et al. (2020) (4)**	Mexico, Sierra Leone, Liberia, Nigeria, Somaliland Ukraine, Georgia Armenia, Kyrgyzstan	Descriptive account	11 academic institutions.	mhGAP-IG content was integrated into academic institutions' pre-service training curricula.	Self-report questionnaire on: institutional background, preparation and adaptation (reasons for using the mhGAP-IG, financial support, adaptation, piloting, obstacles, mode of integration, trainers), teaching process (type of trainees, faculty involved, mhGAP-IG modules used, duration of training, resources and teaching methods) and outcomes (total students trained, benefits, facilitating factors, challenges).	5 institutions introduced mhGAP-IG by revising existing curricula. 3 developed new training programmes and 3 did both. Obstacles included lack of financial resources, lack of institutional support and resistance from some faculty members. Most institutions used mhGAP-IG to train medical students; some trained medical interns, residents and nursing students. Trainees showed improved knowledge and skills to manage mental health disorders; most stakeholders were satisfied.
**Villamil-Salcedo et al. (2017) (5)**	Mexico	2 consecutive cross-sectional studies	Collaborative caregivers from 6 PHC centres in Mexico City: GPs, nurses, social workers, psychologist. Dentists also involved.	Collaborative care model:<br>(1) Implementation of K-10 to identify cases.<br>(2) Training of health personnel.<br>(3) Screening of possible cases by GPs using mhGAP-IG-based checklist card, or filter.	Patients screened, patient psychological distress (K-10), health staff satisfaction.	<u>First study</u>: 18 patients with depressive and/or anxiety disorders were interviewed; but cases were not discussed between GPs and psychiatrist. <u>Second study</u>: psychiatrists and GPs jointly interviewed patients and discussed cases. Of 399 evaluated individuals, 39% were diagnosed with depressive disorder. Afterwards, GPs felt more aware of mental health problems and more interested in identifying them in PHC clinics.
**Robles et al. (2019) (6)**	Mexico	Pre-test/ post-test study	60 clinicians in Jalisco.	mhGAP-IG-based training covered depression and suicide, via 4 weeks' remote learning on general introductory topics and 3 days' face-to-face modules using role-plays and practical exercises.	Knowledge (mhGAP questionnaire), readiness for change (RCQ).	Clinicians had adequate knowledge of depression and its treatment pre-training. Their knowledge of suicide risk management and mhGAP approaches increased post-training. Post-training, the number of clinicians at pre-contemplation and contemplation stages decreased and the number at the action stage increased significantly.
**Hughes & Thomson (2019) (7)**	Haiti, Sudan, Somaliland, India, Myanmar	Descriptive account	<u>Haiti</u>: PHC staff after the 2010 earthquake<br><u>Sudan</u>: health workers in Gezeira State in 2011.<br><u>Somaliland</u>: medical and nursing undergraduates since 2011.	<u>Sudan</u>: mhGAP training was followed by 1 year's remote health worker supervision by a UK psychiatrist.<br>This experience was used to inform similar training programmes in India and Myanmar.<br><u>Somaliland</u>: mhGAP was included in the undergraduate medical and nursing curriculum in 2 universities, supplemented by online distance learning. Postgraduate doctors received training for trainers.	N/A	The authors list opportunities and challenges to implementing mhGAP and their learning points (e.g. ongoing supervision, medication supplies and coordination are essential).

Authors	Country	Study design	Sample	Intervention details	Evaluation details, if any	Summary of findings
**Reginald Fils-Aime et al. (2018) (8)**	Haiti	Retrospective medical records review	318 patients assessed and treated during the first two years of a mobile clinic in Kas.	Community health workers (CHWs) and non-specialist pharmacists were trained to recognise common mental disorders (CMDs) according to the mhGAP-IG.<br><br>The mhGAP-IG was used to treat neurological disorders by psychiatrists and general physicians.	<u>Clinical data</u>: charting of clinical encounters by health workers, CHW-completed patient registrations, pharmacy lists of dispensed medications.<br><u>Quality improvement</u> questionnaire developed to identify gaps in care.<br>In-depth interviews with CHWs to explore patient recruitment and follow-up processes, positive and negative outcomes among patients, challenges, community perceptions of mobile clinics and ways to improve care provision and follow-up.	The commonest mental, neurological and substance abuse (MNS) disorders were depression and epilepsy.<br>Follow-up rates were higher for patients with bipolar disorder and neurological disorders and lower for patients with depression and anxiety disorders. People with mood disorders showed significant reductions in depressive symptoms.
**Hughes et al. (2016) (9)**	Turkey, Syria	Descriptive account	Healthcare providers for internally displaced persons and refugee communities across 60 PHC centres.	Training of trainers based on the mhGAP-IG, where supervision and training systems were developed. Training modules were country specific and training included theoretical and supervisory components (pair work, group work, role-plays, videos).	Number of participants, change in knowledge test scores; feedback on the programme.	<u>Turkey</u>: 16 psychiatrists and other doctors trained, >10% increased knowledge scores, positive course feedback.<br><br><u>Syria</u>: 30 psychiatrists, psychologists, social workers trained, >20% increased knowledge scores, positive overall feedback.
**Karaoğlan Kahiloğulları et al. (2020) (10)**	Turkey	Pre-test/ post-test study	1468 Syrian and Turkish doctors based in 7 refugee health training centres between 2016 and 2019.	mhGAP-IG training was adapted for Turkey. Introductory, essential care and practice, depression, psychosis, child and adolescent, dementia, self-harm/suicide and stress related disorders were selected. 2 additional modules (mental health promotion and anxiety disorders) were added.	Training feedback (online survey), pre-post knowledge tests (mhGAP manual question bank), mean cases identified by 200 trained doctors (Ministry of Health Information System); compliance assessment (compliance with mhGAP-IG guidelines, assessed during mhGAP training using a competency assessment form) and patients' perceived mental health status and satisfaction with services (exit interviews).	Feedback was positive but respondents recommended an additional mhGAP refresher course. Post-training knowledge increased by 9% for Syrian and 5% for Turkish doctors. The mean number of cases identified increased by 27% post-training. Most doctors complied with guidelines. Exit interviews found 95% satisfaction with services; 92% reported having their needs met. Improvements to prescribing and treatment of certain disorders were recommended.
**Kerbage et al. (2020) (11)**	Lebanon	Qualitative study	60 practitioners/ policymakers and 25 Syrian participants involved with mental health services provided for refugees by 10 NGOs.	mhGAP-based training offered to social workers to improve screening and detection of mental health disorders among refugees and enable referrals to clinical services.	Subjective experiences and meanings of distress, well-being, and mental health interventions based on the explanatory model approach to illness and health.	Refugees viewed their distress as a normal shared reaction to adversity, while professionals perceived it as symptomatic of mental illness. Practitioners viewed Syrian culture as an obstacle to providing care and prioritized educating refugees about mental health conditions. Policymakers justified short-term interventions in relation to the emergency setting, while refugees requested community interventions.
**Van Ommeren et al. (2015) (12)**	Jordan	Descriptive account	90 general health workers at 21 PHC centres in 4 cities.	Health workers received mhGAP base course training, followed by monthly supervision and follow-up.	N/A	There are plans to scale up mhGAP training to three other governorates.

Authors	Country	Study design	Sample	Intervention details	Evaluation details, if any	Summary of findings
**Sharifi et al. (2019) (13)**	Iran	Protocol: Randomised controlled trial	GPs treating children/ youth aged 5-15 years with 1 accompanying parent for non-urgent medical, emotional or behavioural concerns.	<u>Intervention group</u>: 2.5 days' mhGAP-IG-based training to help GPs identify emotional and behavioural problems, engage families with care, provide first-line interventions and refer. Training included role plays and case discussions with an in-person booster session 2 months later. <u>Control group</u>: 1 day youth mental health refresher course. All GPs will receive support, feedback and informal consultation from mental health centre staff.	<u>Primary outcomes</u>: total problems score on the SDQ. <u>Secondary outcomes</u>: child/youth functioning, parents' mental health and emotional state (SDQ); parents' functional status (EuroQol EQ-5D-5L, parent satisfaction. <u>Implementation outcomes</u>: fidelity, proportions of children treated in each arm, GP confidence and attitudes towards mental health management (Physicians' Belief Scale).	N/A
**Spagnolo et al. (2018) (14)**	Tunisia	Qualitative study	18 trained PHC physicians in Greater Tunis	mhGAP-IG training included introductory modules, depression, psychosis, self-harm/suicide, alcohol/drug use disorders. Weekly training for 5 weeks included lectures, role plays and discussions facilitated by trainer-psychiatrists.	Interviews to explore contextual factors (structural, organisational, provider, patient, and innovation) that could have influenced the training's anticipated outcomes.	Contextual factors and training influenced knowledge about symptoms of mental illness, knowledge of and confidence in providing treatment, negative beliefs and understanding of PHC staff roles in mental healthcare. Post-training, participants felt uncomfortable with some aspects of treatment for mental health disorders.
**Spagnolo et al. (2019) (15)**	Tunisia	Descriptive account	112 primary care physicians randomised to receive training at two different times. 18 primary care physicians interviewed about the programme.	As above.<br><br>Tutors (primary care physicians with more mental health training) supported trainees during and post-training.	Lessons learned from the implementation and evaluation of the mhGAP-IG in Tunisia.	Political commitment to mental health facilitated the development of partnerships, implementation of the training programme, and its evaluation. Piloting the programme helped to identify challenges attributed to the training program and its implementation, the mental health care system in the Greater Tunis area, and the research tools. Sharing research findings collaboratively helped ensure their validity and encouraged greater knowledge uptake.
**Spagnolo et al. (2020) (16)**	Tunisia	Multi-design including randomised controlled trial	112 primary care physicians in Greater Tunis.	As above.	Knowledge (mhGAP knowledge questionnaire), attitudes towards mental illness and mental health care (MICA-4); self-efficacy (questionnaire); practice (referrals to specialist mental healthcare and importance allocated to mental health per week).	Training had a statistically significant short-term impact on mental health knowledge, attitudes and self-efficacy scores, but not on self-reported practice, maintained at 18 month follow-up. PHC physicians reported a decrease in referrals to specialist services 18 months after training.
**Kamara et al. (2017) (17)**	Sierra Leone	Descriptive account	14 Non-specialist nurses and over 100 physicians, nurses and auxiliary staff at Connaught Hospital, Freetown	General, HIV and epilepsy staff received mhGAP-IG-based training. Clinical staff attended mental wellbeing workshops addressing stigma, stress management, self-care. mhGAP-IG was used during case-based supervision.	Monthly monitoring and evaluation data collected manually from clinic ledgers.	From March to February 2016, 143 patients were seen at the mental health clinic. 20 had survived or had relatives affected by Ebola. Half the patients were diagnosed with mild distress or depression, anxiety disorders, grief or social problems.<br>30 patients presented with psychosis requiring medication.

Authors	Country	Study design	Sample	Intervention details	Evaluation details, if any	Summary of findings
**Echeverri et al. (2018) (18)**	Cameroon, Chad, Democratic Republic of Congo (DRC), Ethiopia, Kenya, Uganda, Tanzania	Process evaluation	Physicians, clinical officers, nurses, psychologists, community staff.<br>4-5-day training: Cameroon (n=97), Chad (n=105), Ethiopia (n=66), DRC (n=51), Kenya (n=76), Uganda (n=100), Tanzania (n=100).<br>Training of Trainers: Cameroon (n=24), Chad (n=25), Ethiopia (n=16), Uganda (n=11).	mhGAP-HIG-based training was held in Cameroon, Chad, and Ethiopia in 2015 and DRC, Kenya, Uganda, and Tanzania in 2016.<br><br>Training of Trainers and Supervisors sessions took place in Cameroon, Chad, Ethiopia, and Uganda.<br><br>Courses paid addressed collaborative working based on a multilevel intervention approach and context-specific referral systems, in addition to mhGAP-HIG content.	The authors reviewed facilitators' reports, disseminated questionnaires to trainees, conducted telephone interviews with UNHCR public health officers and reviewed UNHCR information system data.<br><br>They identified participant-reported changes to practice post-training (e.g. attitudes towards people with MNS disorders, numbers of patients identified and treated, assessment, diagnostic and management skills, improved coordination between providers and stakeholders, increased mental health awareness in the refugee setting) were evaluated.	Facility-and community-based staff reported improved capacity to deliver mental health and psychosocial support interventions and changes in their attitudes towards people with MNS disorders. Service delivery and collaboration among different intervention levels had improved.<br><br>The scarcity of specialist staff in humanitarian settings hindered the establishment of supervision mechanisms.
**Iheanacho et al. (2014) (19)**	Nigeria	Pre-test/ post-test study	83 pre-registration nursing and medical students in their final training year.	4-day mhGAP-IG-based course reviewing mental disorders and treatment, through 1 hour interactive sessions and role plays.	Attitudes toward people with MNS disorders and perceptions of those disorders (FABI, CAMI, and WPA stigma and discrimination questionnaire).	Attitudes favouring socialising with and normalising the lives of people with MNS disorders improved and learners gained understanding of biopsychosocial perspectives on mental illness.
**Ryan et al. (2020) (20)**	Nigeria	Case study	19 community psychiatric nurses (CPNs)<br>48 community health extension workers in Benue state.	mhGAP-IG training was included in Nigeria's Mental Health Policy on Primary Care. mhGAP-IG-trained and supervised CPNs and health extension workers were posted in 1 health centre per local government area.	4 field visits (desk reviews and observations) to collect information on the programme's context, history, model, interventions, organisation, beneficiaries and information system. Interviews with the Project Coordinator, community mental health Project Officer, self-help group development Project Officer and 6 CPNs.	Phase 1 established 45 new mental health clinics in primary care facilities across Benue.<br>13,785 clients were referred to PHC or community -based rehabilitation mental health services.<br>Epilepsy and psychosis were the commonest presentations.
**Ali et al. (2012) (21)**	Sudan	Pre-test/ post-test study	Cohorts of PHC physicians enrolled in the University of Gezira family medicine programme.	1-week mhGAP-IG-based training course delivered to cohorts of PHC physicians weekly for 7 weeks. Teaching methods included small-group problem-based learning, role-plays, mini-lectures, quizzes, clinical interviews with in- and out-patients at 2 local hospitals.	Course evaluation, pre- and post-intervention attitude surveys, knowledge and aptitude tests, including 11 clinical vignettes followed by short-answer questions.	150 PHC physicians were trained. Mean knowledge test scores increased from 19 to 45% post-course. Knowledge of delirium, school refusal and postpartum psychosis increased most, post-course. 95% of respondents rated the course as good or better.
**Aboaja et al. (2015) (22)**	Sudan	Pre-test/ post-test study	500 non-specialist healthcare workers<br>13 out of 17 GPs completed the programme.	GPs attended online E-Supervision support sessions, comprising 1-hour discussions of mental health topics from the mhGAP-IG and clinical cases from their practice.	Pre-course and follow-up assessments at 6 months assessed feasibility of e-supervision, knowledge (mhGAP-IG questionnaire), confidence in managing MNS disorders and beliefs about mental health (questionnaire).	Quantitative and qualitative results showed increased confidence in managing MNS disorders but less improvement in knowledge scores. Attitudes towards mental health improved non-significantly. Supervising psychiatrists noted educational benefits. Technical and cultural differences were challenges.

Authors	Country	Study design	Sample	Intervention details	Evaluation details, if any	Summary of findings
**Osman et al. (2020) (23)**	Sudan	Descriptive account	400 PHC physicians.	mhGAP-IG training to diagnose and treat CMDs.	N/A	400 trained by the scheme, supported by Ministry of Health, Gezira University and WHO local office.
**Gavaghan et al. (2014) (24)**	Somaliland	Pre-test/ post-test study	Undergraduate medical students at Amoud and Hargeisa universities in 2012 (n=36 + 1 nursing student) and 2013 training (n=52).	9-day mhGAP-IG-based course integrated into undergraduate medical degree.<br>Teaching included lectures, role play and case-based discussions.	Pre- and post-course knowledge (mhGAP questionnaire), training satisfaction (ability to understand, usefulness, appropriateness).	Ability to understand the mhGAP-IG was rated 'excellent' by 39% in 2012 and 75% in 2013. The training's usefulness was rated 'good' by 82% in 2012 and 94% in 2013. The course's relevance to Somaliland was rated 'excellent' by 42% in 2012 and 49% in 2013. The range of knowledge scores increased from 28 to 84% pre-course to 68 to 100% post-course.
**Murphy et al. (2017) (25)**	Somaliland, UK	Pre-test/ post-test study	Amoud and Hargeisa university students who had received previous mhGAP-IG teaching and UK students of any year.	Pairs of UK and Somaliland students were matched to complete online problem-based learning tutorials structured around mhGAP-IG modules including affective disorders, psychosis, self-harm and suicide.	Attitudes towards psychiatry (ATP-30); qualitative surveys about programme experience.	Median ATP-30 scores for Somaliland and UK students improved significantly post-participation. Students valued peer connectivity and learning about cultural and psychosocial differences in their partner's country. Somaliland students were motivated by clinical learning and UK students were motivated by global health education.
**Ayano et al. (2017) (26)**	Ethiopia	Pre-test/ post-test study	94 PHC professionals: 44 health officers, 22 diploma nurses, 28 nurses.	5-day mhGAP-IG-based training on 4 priority disorders (alcohol use disorders, depression, psychosis, epilepsy), with supervision and e-mentoring.	Identification of common priority MNS disorders such as psychosis, depression, epilepsy, and alcohol use disorders (WHO case vignettes), knowledge and attitudes towards mental ill-health (19-item questionnaire), clinical case identification.	Knowledge, attitudes and practice improved for all 4 priority disorders. Knowledge increased for psychosis (53%), depression (43%), epilepsy (19%), and alcohol use disorders (54%). Attitudes improved for psychosis (55%), depression (41%), epilepsy (36%) and alcohol use disorders (44%). Case identification increased for psychosis (63%), depression (55%), epilepsy (21%) and alcohol use disorders (41%).
**Catalao et al. (2018) (27)**	Ethiopia	Qualitative study	13 service users and 3 caregivers in Sodo district.	PHC staff received 1 week's mhGAP-IG-based training through PRIME, which developed and implemented capacity building and decision support in health facilities. Staff received mhGAP-IG pocket guides, posters and supervision.	Interviews followed a topic guide with questions on acceptability, satisfaction, barriers to access care, pathways through care and impact of services, focused on epilepsy.	Locating services in PHC decreased transport costs. Experience of services was associated with willingness to promote them to others. Most respondents continued to see traditional healers alongside biomedical care. Most care focused on medication rather than managing epilepsy and its effects. Caregivers and service users described emotional and financial burdens and lack of support. Most reported clinical improvement on medication: over half could return to work.
**Selamu et al. (2019) (28)**	Ethiopia	Cohort study	136 government PHC staff in Sodo district who received mhGAP-IG training, with at least 3 month's experience in that or a similar setting.	Faculty and community-based health workers received training guided by the mhGAP-IG.	Primary outcome: burnout (Maslach Burnout Inventory, human service survey version).<br>Secondary outcome: (at baseline and 6month follow-up): professional satisfaction (JSQ).<br>Exposure variables: socio-demographic/work characteristics, depression (PHQ-9), social support (OSSS-3), stressful life events (LTE), alcohol (AUDIT), work factors (JCQ).	Burnout levels reduced non-significantly at follow-up. High depression symptoms, 2+ stressful life events, being a community health extension worker rather than a facility-based healthcare worker, perceived job and older age were significantly associated with higher levels of emotional exhaustion.

Authors	Country	Study design	Sample	Intervention details	Evaluation details, if any	Summary of findings
**Mutiso et al. 2018 (29)**	Kenya	Cross-sectional study	Trainees: 60 CHWs, 59 traditional healers (THs), 51 faith healers (FHs) and 40 nurses or clinical officers across 20 facilities.<br><br>Community sample: 15,078 participants.	A 5 full-day residential training course using the mhGAP-IG, delivered by a clinical psychologist, PHC physician and nurse. Training was delivered to 3 sub-groups: (1) THs and FHs, (2) CHWs and interested community members, (3) nurses and clinical officers. Training used lectures, case discussions, small group activities, mock screenings and referrals. Individuals screened positive for a mental disorder were assessed using the MINI Plus and referred to the health facility.	N/A	81% of the sample screened positive for a mental disorder, of whom 68.5% attended independent diagnostic assessment.<br><br>Positive predictive value varied between healthcare providers and across disorders, but concordance between primary care mhGAP screen and MIN Plus diagnosis was over 80%.
**Mutiso et al. (2019) (30)**	Kenya	Pre-test/ post-test study	3267 people from a community that had received mental health services from 20 health facilities.	5 day mhGAP-IG training was delivered as described in Mutiso et al. (2018).(29)<br>Research assistants, nurses and clinical officers also received mhGAP-IG training.	Population mental health (MAKS) and how it relates to socio-demographic characteristics and DSM-V/ICD-10 diagnoses.	Mental health-related knowledge increased significantly on post-test scores. Knowledge increases varied according to socio-demographic characteristics including sex, marital status, education, employment status and wealth index.
**Mutiso et al. (2019) (31)**	Kenya	Qualitative study	205 participants: 19 focus group discussions for CHWs, THs, FHs and patients + 10 key informant interviews + 3 key informant discussions with nurses and clinical officers.	CHWs, THs and FHs received mhGAP-IG-based training on CMD screening, in separate cadres. Training included referral to mhGAP-IG-trained nurses and clinical officers.	Interviews and focus groups explored understanding perceptions of the mhGAP-IG model and its impact.	Health providers reported greater job satisfaction, capacity building and interest in mental health training. Patients reported improved mental and physical health, lifestyle, social functioning, family productivity and increased mental health awareness. Improved effectiveness and efficiency of mental health service delivery and increased referrals to PHC were identified.
**Mutiso et al. (2019) (32)**	Kenya	Pre-test/ post-test study	2305 people from 20 health facilities across Makueni Country who had received community mental health services.	1-hour community education sessions based on the mhGAP-IG were offered to enhance mental health knowledge and awareness. Sessions included priority MNS disorders. Adult mhGAP-IG modules were used to train clinicians, CHWs, THs and FHs. Participants were trained to use the mhGAP-IG screening tool.	Outcomes included: socio-demographic and wealth index questionnaire (adapted from World Bank International wealth index), mental disorder diagnoses (MINI-Plus) and attitudes (DISC-12).	60% of patients were followed up at 6 months. 'Reported/experienced discrimination' declined significantly post-intervention. Multivariate linear mixed model regression found that reduced 'unfair treatment' scores were associated with depression, low education, marriage, youth, self-employment and wealth. For the 'overcoming stigma' domain, better outcomes were associated with depression, male sex, education, employment, wealth and was associated with better outcomes.

Authors	Country	Study design	Sample	Intervention details	Evaluation details, if any	Summary of findings
**Akol et al. (2018) (33, 34)**	Uganda	Randomised controlled trial	36 PHC clinics (18 per arm) in Mbale and Sironko districts.	Intervention group: 2 PHC workers per cluster clinic were trained over 5 days to screen and refer child and adolescent MNS disorders using the mhGAP-IG. Trainees received materials on screening using the mhGAP-IG, for reference. Control group: no intervention.	Primary outcome: proportion of intervention arm clinics relative to control arm clinics recording at least 1 child and adolescent mental health case over 3 months' follow-up. Secondary outcome: likelihood of child/ adolescent patients receiving a non-epilepsy mental health diagnosis in the intervention group compared with the control group.	Nearly two thirds of all clinics identified and recorded at least 1 child/adolescent mental health diagnosis out of 40,692 child clinic visits over 4 months, compared to 28% pre-intervention. The odds of identifying and recording a child or adolescent mental health diagnosis were 2.5 times higher in the intervention than control groups at 3 months' follow-up.
**Akol et al. (2017) (35)**	Uganda	Pre-test/ post-test study	36 clinical officers, nurses and midwives at 18 randomly selected primary healthcare clinics in eastern Uganda.	5-day training on child and adolescent mental health screening and referral was offered to clinical officers, nurses and midwives. Training used the mhGAP-IG and International Association of Child and Adolescent Psychiatry and Allied Professions-produced slides.	Participant child and adolescent mental health knowledge scores pre- and post-training, both generally and as a result of training.	33 participants completed pre- and post-training measures. Clinical officers and nurse/midwives showed increased mean knowledge scores post-training. Clinical officers had significantly higher knowledge scores than nurses and midwives but cadre was not significantly associated with improvements in child and adolescent mental health knowledge.
**Ahren et al. (2020) (36)**	Malawi	Pre-test/ post-test study	Non-specialist health workers working in 5 districts in southern Malawi.	A 4 day mhGAP-IG-based training course adapted for Malawi was delivered to district mental health teams. Modules included psychosis, depression, alcohol and substance use disorders. A 2 day refresher course was delivered 6 weeks later, with monthly supervision for 3 months.	At baseline and 6 months' follow-up: self-reported knowledge (mhGAP-IG test), confidence, and attitudes (Community Attitudes to the Mentally Ill Scale). Case detection: inspection of hand-written case registers. Acceptability of training via focus groups with trainees, service users and carers.	Non-specialist healthcare workers' knowledge and confidence scores significantly increased post-training, compared to pre-training. Increased scores were maintained at 6 months' follow-up. No statistically significant changes in attitude scores was detected. Case detection rates increased immediately post-training, compared to pre-training. The programme was acceptable to trainees.
**Kokota et al. (2020) (37)**	Malawi	Pre-test/ post-test study	43 PHC workers from 18 PHC clinics serving Mulanje province.	A 2 day mhGAP-IG-based training course was offered to PHC workers, including psychosis, depression, alcohol and substance use disorders. Training employed presentations, tutorials, videos, case-studies and role-plays. Support and supervision continued through monthly outreach visits.	Attitudes (CAMI); knowledge (mhGAP knowledge questionnaire); and confidence (instrument previously used in Malawi).<br><br>Numbers of new diagnoses of MNS disorders was collected by clinical registrars at all 18 health centres.	The mean knowledge score increased significantly immediately post-training compared to pre-training and was sustained at 6 months' follow-up. The mean confidence score also increased significantly immediately post-training compared to pre-training and was sustained at 6 months' follow-up. A significant increase in mental health service utilisation was identified post-intervention.
**Patel et al. 2019 (38)**	Zambia	Pre-test/ post-test study	9 clinical officers (from an original 10) across 4 PHC centres.	Trainees received a 3 week structured course on paediatric seizures and epilepsy, informed by the mhGAP-IG.	Knowledge of paediatric epilepsy.	Pre- and post-course assessments showed improved knowledge about medical management of epilepsy, recognition of focal seizures, improved history taking and medication titration. However, learning about provoked seizures, use of diagnostic studies and aetiologies of epilepsy was limited. The authors identified improvements to roll out training to a wider selection of PHC staff.

Authors	Country	Study design	Sample	Intervention details	Evaluation details, if any	Summary of findings
**Patel et al. 2020 (39)**	Zambia	Pre-test/ post-test study	23 participants (from an original 24) across 3 sites (48% clinical officers, 43% nurses, 9% other).	Following the previous pilot implementation, a standardised paediatric seizure history and assessment form and validated diagnostic questionnaire were developed. A 5 day mhGAP-IG-informed course comprised interactive and case-based teaching, videos, 2 supervised clinical sessions, 2 role plays and case discussions.	Knowledge of paediatric epilepsy.	Pre- and post-course assessments showed significant improvement in 7/15 knowledge domains.<br><br>Chart reviews showed significant improvements in documentation of seizure descriptions, frequency and suspected aetiology,
**Sibeko et al., 2018 (40)**	South Africa	Pre-test/ post-test study	58 CHWs supervised by 4 NGOs in the Western Cape.	Training informed by the mhGAP-IG.	Knowledge (case vignettes and MAKS), confidence in delivering mental health support (MHNCCS), personal attitude towards mental illness (CAMI); acceptability and feasibility (daily evaluation questionnaires and a training questionnaire).	Most participants demonstrated significant knowledge improvements sustained at 3 months' follow-up. Confidence and some attitudes improved significantly but authoritarian attitudes were unchanged.<br>The training was considered acceptable and feasible.
**Gumber et al. 2015 (41)**	Sri Lanka	Pre-test/ post-test study	40 doctors working as medical officers for mental health or psychiatry registrars (higher trainees) at the National Institute of Mental Health (NIMH), Colombo.	A 5 day pilot of mhGAP training modules on intellectual disability (mhGAP-ID) comprising lectures, role-play, audio-visual aids and reflection on clinical practice. Case scenarios allowed trainees to practise responding to belief in alternative medicines and religious cures. Learning resources were shared prior to the course.	Knowledge of intellectual disability on multiple-choice questions (MCQs).	Mean knowledge test scores improved from 77 to 84% correct, post-course.<br><br>The mean course satisfaction rating was 85%.<br><br>Learners recommended additional content about counselling parents, sexual relationships and marriage, and childbearing for people with intellectual disability.
**Doherty (2017) (42)**	Sri Lanka	Trial registration: stepped wedge cluster randomised trial	Community representatives and PHC workforce (doctors, nurses, health service assistants) working at 25 participating centres across 5 northern districts.	<u>Intervention group</u>: Cohorts of PHC staff will receive 3 days' mhGAP-IG-based training at intervals. Staff will use the mhGAP-IG to diagnose, treat and refer patients.<br><u>Control group</u>: untrained PHC staff will continue treatment as usual.<br><u>Community representatives</u> will not be randomized but will use the training to raise awareness.	Knowledge scores (at baseline, 3, 6 and 12 months' follow-up), socio-demographics of PHC attendees, mood, anxiety and trauma symptoms (Hopkins Symptoms Checklist and Harvard Trauma questionnaire).	- N/A
**Humayun et al. (2016) (43)**	Pakistan	Descriptive account	58 PHC staff trained to care for internally displaced people in Bannu teaching hospital.	Monthly mental health camps at for 6 months, where staff were trained with the mhGAP-IG.<br>CHWs also received psychosocial training.	Mental health needs assessment.	Psychiatrists, psychologists, psychiatric nurses and psychosocial workers offered pharmacological and psychological mental healthcare to 680 people who attended mental health camps. 21% returned for follow-up; others were followed up in the community by psychosocial teams.

Authors	Country	Study design	Sample	Intervention details	Evaluation details, if any	Summary of findings
**Pathare et al. (2020) (44)**	India	Protocol: Cluster randomised controlled trial	Adolescents in grade 9 at public health high schools.<br><br>Adult community members (18+ years) living in 124 villages in Mehsana district.	Intervention group: integrated suicide prevention programme: (1) Secondary-school-based intervention, (2) Community intervention to reduce access to pesticides from storage facilities, (3) CHW training in recognition, management and referral of suicidal people using the mhGAP-IG<br>Control group: enhanced usual care (leaflets with information on available mental health services).	Primary outcome: reduction in suicides and attempted suicides (key informant surveillance system), suicidal ideation (SIDAS); depression symptoms (PHQ-9); staff knowledge (self-reported questionnaire), attitudes towards suicide prevention, skills (WHO mhGAP questionnaire), translation into practice (staff diaries).<br>Secondary outcomes: process measures (log schools approached and agreeing), focus groups, costing.	- N/A
**Dyer & Biswas (2019) (45)**	Bangladesh	Descriptive account	Médecins Sans Frontières team.	mhGAP-IG training implemented alongside a peer supervision network and individual supervision.	N/A	664 patients with moderate to severe disorders were seen post-training. The commonest presentations were psychotic disorders.<br>Training a doctor and medical assistant from NCD team is planned.
**Momotaz et al. 2019 (46)**	Bangladesh	Descriptive account	Initial: 8 physicians, 7 refugee camp staff, 6 psychosocial staff. Refresher training; 10 government facility physicians, 3 refugee camp physicians, 6 psychosocial staff.	3 day initial and refresher courses covered 7 mhGAP-IG and 2 mhGAP-HIG modules. Training used locally accepted terminology, discussed cultural context and clinical reality. Refresher training introduced the mhGAP-IG mobile phone app. 3 day supervision visit from trainers and a psychiatrist 1 month later.	N/A	Over 75% were 'very confident' to apply the training to their work. 6 participants who attended both courses reported improved attitudes towards people with mental health problems.<br>Supervision visits rated most participants as average in for psychosocial intervention and referral skills.<br>7 physicians reported delivering 142 mental health consultations in the month post-training.
**Chase et al. (2018) (47)**	Nepal	Case study	10 governmental and non-governmental stakeholder groups involved in mental health system development	Mental health awareness raising activities: disseminating 'Information, Education, and Communication' materials on mental health, community theatre performances, radio programs, anti-stigma campaigns, and the construction of billboards on mhGAP priority disorders.<br>Prescribers were trained in the mhGAP-IG.	Review of 168 documents from grey literature on the mental health and psychosocial response to the earthquake (collected through online information-sharing platforms and response coordinators).<br>Focus group discussions with key stakeholders.	Key achievements post-earthquake included training PHC staff in affected districts using the mhGAP-IG and training new psychosocial staff, appointing government and WHO Country Office mental health focal points, adding new psychotropic drugs to the government's free drugs list, a community mental healthcare package and training curricula for different health worker cadres and revising mental health plans, policy, and financing mechanisms. Remaining concerns: government ownership and financing are insufficient to sustain services.

Authors	Country	Study design	Sample	Intervention details	Evaluation details, if any	Summary of findings
**Rai et al. (2018) (48)**	Nepal	Qualitative study	17 key informants (service users and caregivers).	mhGAP-IG training and use is being piloted as part of a broader package of care. The study evaluated integrating service users as co-facilitators for mhGAP-IG and psychological intervention training, as a stigma reduction initiative.	Interviews with caregivers focusing on service users' experiences of treatment processes, involvement in training, barriers to participation and stigma.<br>Interviews with service users focused on the impact of programme participation on their families and how caregivers facilitated or obstructed their participation.	Caregivers' perceived benefits of service user involvement in mhGAP-IG training included reduced caregiver burden, learning new skills and opportunities to develop support groups. Barriers included time constraints, caregivers' fear of worsening stigma and lack of trust between caregivers and service users. This could be mitigated through family engagement. More attention to orientating caregivers to the programme was required, as it reduced stigma in families.
**Kohrt et al. (2018) (49)**	Nepal	Protocol: feasibility study	150 PHC workers and 100 patients across 34 primary care centres in Chitwan	Intervention group: control group training plus training service users in recovery as co-facilitators.<br>Control group: standard 10 day PRIME training based on the mhGAP-IG with psychosocial modules on communication skills, supportive techniques, and health education. Psychological treatments available include the Healthy Activity Program and Counselling for Alcohol Problems.	Primary outcomes: acceptability and feasibility (qualitative interviews with PHC staff, trainers and service users).<br><br>Secondary outcomes: quantitative information on health worker outcomes, mental health stigma (Social Distance Scale), clinical knowledge (mhGAP questionnaire), clinical competency (ENACT), implicit attitudes (IAT), patient outcomes including stigma-related barriers to care, daily functioning and symptoms.	N/A
**Kohrt et al. (2018) (50)**	Uganda<br>Liberia<br>Nepal	Pre-test/ post-test study	206 health workers in humanitarian settings impacted by political violence, Ebola and natural disasters	Psychiatrists and psychosocial experts trained non-specialist PHC workers to identify and treat epilepsy, psychosis/mania and depression using mhGAP-IG. Training addressed reducing stigma and discrimination, community sensitisation, detection, referral, home-based care, support groups and microfinancing activities.	Participants were evaluated for knowledge (mhGAP knowledge test), attitudes (mhGAP package questions, adapted questions from PRIME, MICA, and SDS) and competence (structured role plays with standardised patients, patient-completed ENACT).	Trainees showed increased knowledge and reduced stigma but were only competent in 65% of skills. Although communication skills and empathy were usually adequate, assessing physical and mental health, addressing confidentiality, involving family members in care, and assessing suicide risk required improvement. Higher competency was associated with lower stigma, but not with knowledge.
**Rajbhandari et al. (2019) (51)**	Nepal	Cohort study	Epilepsy field workers in Myadgi district	3-day mhGAP-IG-based training on epilepsy care	Mortality, change in diagnosis (reviewed by neurologist), drug-related side effects (at telephone follow-up), effectiveness (reduced or less frequent seizures or improved self-rated wellbeing), satisfaction (questionnaire).	112 patients with suspected epilepsy were identified and managed over 18 months. 43% were untreated. At follow-up, 1 patient had died due to a non-epileptic cause. Diagnostic agreement at neurologist assessment was 93%. 5% of patients had medication side-effects. Seizures ceased in 33% of patients and reduced in 57%. 96% of patients preferred this service to travelling to other doctors.

Authors	Country	Study design	Sample	Intervention details	Evaluation details, if any	Summary of findings
**Kohrt et al. (2020) (52)**	Nepal	Pre-test/ post-test study	41 PHC providers working in government facilities in Chitwan district	PHC staff received 10-days' mhGAP-IG and psychosocial support plus an anti-stigma intervention based on service user recovery stories and social contact, aspirational figures, myth busing, stigma didactics and collaboration.	Stigma (SDS), knowledge (mhGAP knowledge questionnaire), attitudes (mhGAP attitudes questions), clinical competence (ENACT).<br><br>Focus groups and key informant interviews were used to evaluate the training.	Trainees described altered perceptions of violence and the ability to treat mental illness effectively. Willingness to interact with a person with mental illness increased from 54% pre-training to 81% , 16 months post-training. Observed clinical competence increased from 49% pre-training to 93%, 16 months post-training.
**Li et al. (2015) (53)**	China	Randomised controlled trial	77 CHWs in Guangzhou (40 in 4 regions allocated to intervention, 37 in 4 regions allocated to control)	Intervention group: mhGAP-IG and Chinese Medical Association guideline-based training with supervision as needed.<br>Control group: usual care following standard training.	Pre-course, post-course, 6 and 12 months' follow-up: mental health knowledge (assessment schedule and MAKS), stigma (MICA and RIBS).	Intervention group knowledge scores were higher than the control group post-training and at 6 and 12 months' follow-up. Mean MAKS scores increased more in the intervention than the control group when controlling for age, sex, marital status, title and time. At 6 months, mean MICA scores decreased more in the intervention than the control group. At 6 and 12 months, mean RIBS increased more for the intervention than the control group when controlling for age, sex, marital status, title and time.
**Li et al. (2019) (54)**	China	Cluster randomised controlled trial	293 care assistant workers across 4 districts of Guangzhou (139 randomized to the intervention, 154 randomized to the control group)	Both groups were trained in mental health and the financial assistance policy of care assistant workers.<br>Intervention group: mhGAP-IG-based anti-stigma module added.<br>Control group: First two modules only	Participants were measured before and after training: belief that other people will devalue or discriminate against someone with a mental illness (PDD); attitudes towards mental illness and psychiatry (MICA), and sigma related knowledge and correct identifications of mental illnesses (MAKS).	There were significant lower scores on PDD and MICA scales in the intervention group post-training in comparison with the control group. No significant difference was found on MAKS total score between the two groups post-training. Both groups better identified schizophrenia, depression, and bipolar disorder before and after training.
**De Moraes Weintraub et al. (2016) (55)**	Philippines	Retrospective medical records review	General doctors, nurses and other health workers treating patients at Médecins Sans Frontières mental health services.	Staff were trained to deliver mental healthcare in PHC according to the mhGAP-IG, with 6 months' support and supervision.	Clinical data on patient sex, age, origin, type of referral, type of mental health session, reason for seeking mental health care, mental health symptoms, history of psychotropic medication, psychotropic medication prescribed, and outcomes of care provided.	172 persons sought mental healthcare. Of 134 (78%) people with complete data, 37 (28%) had a severe mental disorder, with psychotic symptoms most common.<br>Psychotropic treatment was prescribed for 33 (75%) people with mental disorders and for 11 with non-severe mental disorders.
**Ryan et al. (2020) (56)**	Philippines	Descriptive account	At least 1 non-specialist in >90% of 159 health units and 32 district and provincial hospitals in Eastern Visayas, plus 30 PHC staff.	mhGAP-IG-based training and access for doctors to psychotropic medication.	N/A	In the 5 months after a deadly typhoon struck the municipality of Guiuan, > 130 people accessed care. Among 37 people diagnosed with a severe mental health disorder, 68% improved sufficiently for discharge or were referred to the regional health unit for further care.

Authors	Country	Study design	Sample	Intervention details	Evaluation details, if any	Summary of findings
**Charlson et al. (2019) (57)**	Fiji	Process evaluation	678 health workers based at 6 Central and 6 Western healthcare settings out of 41 selected from a list of health workers trained between 2014 and 2017.	Healthcare workers were trained using the mhGAP-IG between 2014 and2017	Trainee semi-structured questionnaire for capturing CFIR domains (e.g. mhGAP training and supervision, application to practice and competence).<br>Focus groups explored themes raised in questionnaires, perspectives on the mental health system and governance issues.<br>Medicine audit at the facility level to assess availability of psychotropic medications.	66 participants reported that mhGAP was valuable and easy to use and had reasonable knowledge and willingness to change. The need for improved planning and leadership was identified.
**Setoya & Kestel (2018) (58)**	14 Small Island Developm ent States, (Pacific Ocean), 13 English-speaking Caribbean countries	Descriptive account	>900 doctors and nurses (SIDS) + 28 GPs and nurses from 6 Caribbean countries (first round), 42 participants from 13 Caribbean countries (second round)	SIDS: mental health stakeholders attended an in-country planning workshop, identifying trainers and trainees, supervision systems, drug availability, monitoring and evaluation. For larger countries, mental health specialists received training of trainers before delivering training locally.<br>Caribbean: PAHO hosted online mhGAP training.	Knowledge and confidence ratings.	SIDS: Post-training tests showed improved knowledge, confidence in assessing and managing people with mental disorders. An online course is planned.<br>Caribbean: post-training tests showed increased knowledge and the course received positive feedback. Second and third rounds of the course took place and a round for Spanish-speaking participants in 2016.
**Usher et al. (2014) (59)**	Cook Islands Solomon Islands Palau Kiribati Papua New Guinea Nuie, Fiji	Pre-test/ post-test study	18 Pacific Island health services staff	4 weeks' training followed mhGAP-IG principles, through lectures and invited presentations, workshops, tutorials, exercises and activities.	Self-reported mental health knowledge, skills, and attitudes (Nurses Self Report questionnaire).	Training significantly improved the mental health knowledge, skills, and attitudes of trainees.

Table 5: Summary of included studies describing clinical uses of mhGAP-IG

Authors	Country	Study design	Sample	Intervention details	Evaluation details	Summary of findings
**Miguel-Esponda et al. (2020) (60, 61)**	Mexico	Feasibility study	Quantitative: electronic record-registered patients attending 10 PHC clinics. Qualitative: 2 focus groups + 24 semi-structured interviews with 14 doctors, 13 nurses, 10 clinical supervisors.	mhGAP-IG-based services offered at PHC clinics including case identification, diagnosis, pharmacological treatment, individual and group talk-based interventions and home visits.	Quantitative process indicators extracted from electronic records included amount (dose) and quality (fidelity) of services delivered. Qualitative data: implementation outcomes (penetration, fidelity, acceptability, appropriateness, feasibility).	PHC staff delivered mental health consultations to 486 adults with a mood or anxiety disorder. Programme fidelity was limited: talk-based interventions were provided in 24% of consultations. Only 42% of service users attended >50% of scheduled follow-up consultations, hindering fidelity. Low attendance was partly attributed to limited appropriateness. Acceptability and feasibility were achieved through strong organisational support.
**Gureje et al. (2015) (62)**	Nigeria	Protocol: Pragmatic cluster randomised controlled trial	Women screening positive for depression across 29 clinics offering full maternal and child health services.	Intervention: maternal depression package comprising psychoeducation, problem solving therapy and parenting skills training. The intervention will be delivered by clinic staff using the mhGAP-IG adapted to the Nigerian context. The 3 day training course will comprise didactic lectures, clinical demonstrations and role plays. It will be followed up with 3 days' top-up. Control: enhanced usual care: health workers will receive 2 days' mhGAP-IG-based training on depression.	Primary outcome: depression recovery (EPDS <6) at 6 months postpartum. Secondary outcomes: EPDS score, maternal disability (WHODAS), parenting skills (MAMAs, HOME-IT); stigma experiences (Discrimination and Stigma Scale); health service use (CSRI-PND, adapted from SUQ); infant development (child's cognitive, language, personal-social, fine and gross motor development: Bayley Scales of Infant Development).	N/A
**Gureje et al. (2015) (63)**	Nigeria	Protocol: cluster randomised controlled trial	Participants attending PHC clinics and screened using PHQ-9 and CIDI.	Intervention: mhGAP-IG depression module, contextualised and adapted to the Nigerian health system, delivered by clinic staff, plus Problem Solving Therapy (3 days' training and 2 days' top-up training). Control: enhanced usual care: 2 days' mhGAP-IG-based training minus Problem Solving Therapy.	Primary outcome: depression recovery (PHQ-9<6) at 12 months. Secondary outcomes (at 6 and 12 months): changes in depression (PHQ-9), disability (WHODAS 2.0), quality of life (WHO Quality of Life instrument) and service utilization (SUQ).	- N/A
**Gureje et al. (2017) (64, 65)**	Nigeria and Ghana	Protocol: Randomised controlled trial	296 participants recruited from PHC clinics and neighbouring facility clusters.	PHC staff in both arms received mhGAP-IG training on psychosis. Intervention arm: collaborative approach between PHC providers and complementary therapists. Control arm: enhanced usual care (without collaborative care).	Primary outcome: PANSS improvement 6 months later. Secondary outcomes: 3 and 6 month follow-up: disability (WHODAS 2.0), harmful treatment and victimisation experiences, perceived stigma (ISMI), caregiver burden (FBIS); service use and costs (Service Utilization Questionnaire).	- N/A

Authors	Country	Study design	Sample	Intervention details	Evaluation details	Summary of findings
**Gureje et al. (2019) (66)**	Nigeria	Cluster randomised controlled trial	686 antenatal women attending antenatal care clinics diagnosed with DSM-IV major depression (intervention group, n=234, control group, n=234)	Intervention group: high-intensity treatment comprising basic mhGAP-IG-recommended psychosocial treatment + 8 weekly problem-solving therapy sessions determined by EPDS scores. Control group: low-intensity treatment comprising basic mhGAP-IG-recommended psychosocial treatment	Primary outcome: remission of depression at 6-months postpartum (EPDS score <6). Maternal secondary outcomes: depression (EPDS); disability (WHODAS); parenting skills (MAMA and Infant Toddler version, Home Inventory for Measurement of the Environment), stigma (DISC-12).	6 month follow-up showed remission rates of 70% in the intervention group and 66% in the control group. The intervention was more effective for severe depression and was associated with more exclusive breastfeeding.
**Gureje et al. (2019) (67)**	Nigeria	Cluster randomised controlled trial	35 clinic clusters delivering 24 hour clinical services with regular clinician supervision: 18 allocated to the intervention group (n=1178) + 17 allocated to the control group (n=547))	Intervention group: stepped-care intervention: staff trained to identify and treat depression using the mhGAP-IG and to deliver structured behavioural activation and problem-solving therapy. Training comprised lectures, clinical demonstrations, role plays, mobile phone support and supervision by GPs). Control group: enhanced usual care: staff received 2 day mhGAP-IG training.	Primary outcome: proportion of patients in remission at 12 months (PHQ-9 <6). Secondary outcomes: depression (PHQ-9 score at 3, 6, 9 and 12 months), disability (WHODAS), quality of life (WHOQOL), health-care use (SUQ) at 6 and 12 months.	Similar proportions in each group showed remission of depression.
**Adewuya et al. (2019) (68)**	Nigeria	Cluster randomised controlled trial	Adults with depression across 10 primary care centres (intervention group: n=439, control group: n=456.	Staff were trained to deliver a collaborative care intervention using the mhGAP-IG training manual. Intervention group: mobile telephone supported collaborative stepped care group (e.g. text messages to remind patients about appointments, medication adherence, relapse symptoms, automated messages after missed appointment and calls from CHWs about missed rescheduled appointments). Control group: collaborative stepped care group without telephone support.	Primary outcome: intervention adherence at 6 and 12 months follow-up (attendance logs). Secondary outcomes: depression recovery (PHQ-9 <6), disability (WHODAS score >12), quality of life (WHOQOL-brief overall score >3), number of deaths, self-harm incidents, referrals to mental health services, losses to follow-up at 12 months, health economic costs (CSRI).	The intervention group showed significantly better adherence than the control group at 6 and 12 months' follow-up. Compared to the control condition, the intervention was associated with significantly more recoveries, better quality of life, longer retention in treatment, was more cost effective and more acceptable to patients.
**Adewuya et al. (2019) (69, 70)**	Nigeria	Cluster randomised controlled trial	907 patients from 10 primary care centre (intervention: n=456, control: n=451).	Intervention: stepped care: psycho-education, Problem-Solving (PST-PC) or antidepressants. All PHC staff attended 5 days' mhGAP-IG-based training and a 2 day refresher course 4 weeks later. Training included psychosis, suicidality and referring to specialists. Control: psychoeducation and leaflet about depression.	Primary outcome: Recovery at baseline and 12 months' follow-up (PHQ-9). Secondary outcomes: Recovery at 4 and 6 months' follow-up, disability (WHODAS), improved quality of life (WHOQOL-brief overall), intervention adherence, deaths, self-harm incidents, referral to mental health services at 12 months' follow-up (attendance logs).	At 12 months, clinical recovery was significantly higher in the intervention group than the control group (60% versus 18%). The intervention group had significantly better quality of life and less disability, death, and fewer self-harm incidents compared with the control group.

Authors	Country	Study design	Sample	Intervention details	Evaluation details, if any	Summary of findings
**Gureje et al. (2020) (71)**	Nigeria	Protocol: Randomised controlled trial	Newly registered adolescent women at antenatal care clinics across 11 local government areas of Ibadan, scoring 12 or more on the EPDS.	Intervention group: mhGAP training followed by 3 days' training in behavioural activation, problem-solving therapy and parenting skills. Training included a 1 day refresher course 3 months later. The 'neighbourhood mother' component provided social support. Control group: treatment as usual: staff previously trained to use mhGAP-IG.	Primary outcome: depression symptoms (EPDS), parenting skills (HOME-IT). Secondary outcomes: depression remission (EPDS <6), disability (WHODAS 2.0), attitude and adjustment to pregnancy and motherhood (MAMAS), quality of life (WHOQoL-BREF), mother-infant interactions (PCQ), family planning and new pregnancy questionnaire, social support (PICSS), infant weight, height, birth head circumference, nutrition.	- N/A
**Hailemariam et al. (2019) (72)**	Ethiopia	Cross-sectional study	65 health extension workers; 26 key community informants (community-based data collectors, community leaders).	Health extension workers and key informants were trained to diagnose psychosis and bipolar affective disorder using the mhGAP-IG.	Access to care: attendance at PHC for probable SMI. Non-engaged group: PSQ in patients who did not attend their PHC referral appointment. Barriers to accessing care (BACE-3, SEMI). Engaged group: people who attended PHC assessed to confirm diagnosis with OPCRIT Both groups: disability (WHODAS 2.0), social support (OSSS-3), alcohol use (AUDIT), discrimination experiences (DISC-12), physical and sensory impairment (BPIRC).	The new service's contact coverage was estimated as 81%. Rural residents had 3.8x the odds of not accessing care, in part due to geographical distance from health facilities. Greater functional impairment was associated with higher odds of engagement. The most frequently endorsed barriers to engagement were thinking the problem would get better by itself and concerns about the cost of treatment.
**Habtamu et al. (2019) (73)**	Ethiopia	Case-control study	2038 adults attending 8 PHC centres across Sodo district. Patients screening positive for depressive symptoms (PHQ-9 >5) were compared with matched controls.	PHC clinicians received 10 days' training to diagnose depression using an adapted mhGAP-IG version: 5 days using training materials and 5 days in psychiatric outpatient clinics, with regular psychiatric nurse supervision.	Disability (WHODAS 2.0), depression (PHQ-9), suicidality (CIDI), social support (OSSS-3), problematic alcohol use (AUDIT), clinical diagnosis of depression.	There was no significant difference between patients with and without depression diagnoses. Clinician-confirmed cases had more severe symptoms, suicidality and lower social support than non-diagnosed patients. Functional impairment was associated with depression severity and less social support. Diagnosed cases had more functional impairment than controls.
**Forthal et al. (2019) (74)**	Ethiopia	Cross-sectional study	300 people diagnosed with SMI and their caregivers.	Health extension workers and community key-informants identified patients with SMI. They were referred to local PHC services and evaluated by nurses or health officers trained with the mhGAP-IG to diagnose and treat people with psychosis or bipolar affective disorder.	Primary outcome: discrimination experiences (DISC-12). Primary exposure: rural/urban area. Potential confounders: clinical diagnosis (OPCRIT), severity (BPRS-E), disability (WHODAS 2.0), alcohol (AUDIT), social support (OSSS-3) poverty.	63% had experienced past-year discrimination. Urban residents were 1.7 times more likely than rural residents to experience discrimination: unfair treatment from friends, police, in keeping a job, and in safety. Urban residents had significantly higher DISC-12 scores than rural residents.

Authors	Country	Study design	Sample	Intervention details	Evaluation details, if any	Summary of findings
**Hanlon et al. (2019) (75)**	Ethiopia	Uncontrolled study	300 people diagnosed by with SMI by trained PHC staff.	People with possible SMI were referred to the nearest PHC clinic to be assessed. Staff had received 5 days' mhGAP-IG training and 5 days' practical training at a psychiatric clinic. Staff used the mhGAP-IG to assess referrals, diagnose SMI and initiate treatment.	Primary outcomes: symptom severity (BPRS-E), disability (WHODAS 2.0) Secondary outcomes: experience of discrimination (DISC-12 unfair treatment sub-scale), self-reported restraint, chaining or confinement), alcohol use (AUDIT), depression (PHQ-9), suicide attempts (MINI).	95% of PHC worker diagnoses of SMI were verified by psychiatric nurses. All clinical and social outcomes improved significantly following intervention. The impact on disability was greater than the impact on symptom severity. Being restrained reduced from 25 to 11%, and discrimination scores reduced significantly.
**Tirfessa et al. (2020) (76)**	Ethiopia	Case-control study	239 people with SMI and 273 matched control households.	Clinicians were trained to provide care for people with SMI, depression, epilepsy and alcohol use disorders through 10 days' mhGAP-IG training, with at least monthly face to face supervision.	Primary outcome: food security status at 12 months follow-up (HFIAS). Potential predictors and mediators: discrimination (DISC-12); physical impairment (Washington group general disability measure), disability (WHODAS 2.0), work impairment (LIFE-RIFT); symptom severity (BPRS-E).	Maintenance or improvement in food insecurity status was seen in 52% of households with a person with SMI compared to 40% of control households. Food security was indirectly associated with reduced symptom severity via reduced work impairment and discrimination.
**Musyimi et al. (2017) (77)**	Kenya	Qualitative study	Community health workers affiliated to a health facility, FHs affiliated to a place of worship and registered THs.	Staff received 2 days' mhGAP-IG training. The mhGAP-IG master chart provided thresholds to refer patients to PHC providers.	Focus groups explored participants' views about challenges to using the mhGAP-IG, including referring patients with mental disorders.	Challenges included patients' mistrust of informal health providers, cultural misunderstanding and initial stigma towards mental health problems.<br>Resource barriers, treatment resistance and referral system limitations were challenges.
**Musyimi (2017) (78)**	Kenya	Protocol: Cluster randomised controlled trial	Pregnant women recruited from 2 sub-counties (clusters) in Makueni county.	Intervention group: traditional birth attendants for pregnant women will receive mhGAP-IG training and then provide mhGAP-IG psychoeducation Control group: advice to refer patients to the nearest clinic	Primary outcome: reduction in depressive symptoms (EPDS). Secondary outcomes: intimate partner violence, quality of life (WHOQoL), patient satisfaction, suicidality (Beck's Suicidality Scale), disability (WHODAS).	N/A
**Mutiso et al. (2020) (79)**	Kenya	Uncontrolled study	1333 parents from 23 randomly selected primary schools, out of 1791 assessed at baseline.	Parents received psychoeducation on child wellbeing and recognising mental disorders based on the mhGAP-IG.	Primary outcomes: awareness and perception of mental health symptoms in children (CBCL and BMP-P).	Mean CBCL and BMP-P scores increased significantly between at 6 months' follow-up
**Mutiso et al. (2019) (80)**	Kenya	Uncontrolled study	2306 patients from 20 healthcare facilities across Makueni county.<br>40 nurses and clinical officers.	Staff received 5 days' full mhGAP-IG training, followed by face to face supervision every two weeks and virtual supervision on demand.	Confirmed DSM-IV diagnosis following mhGAP-IG screening (MINI-Plus), disability (WHODAS 2.0), quality of life (WHO QoL-BREF), depression (PHQ-9), suicidality (BSS), psychosis (WERCAP), epilepsy, substance use (ASSIST)	All participants received psychoeducation and most received medication. There was a significant reduction in disability, improvement in seizure control and improvement in clinical outcomes following intervention.

Keynejad R, *et al. Evid Based Ment Health* 2021; 24:124–130. doi: 10.1136/ebmental-2021-300254

Authors	Country	Study design	Sample	Intervention details	Evaluation details, if any	Summary of findings
**Kumar et al. (2020) (81)**	Kenya	Protocol: Feasibility study	Target size: 90 pregnant adolescents aged 13-18 years attending 2 mental health clinics in their 1st-2nd trimester, scoring >12 on EPDS.	Intervention group 1: IPT-G Full (8 sessions) in the context of mhGAP mental health service delivery. Intervention group 2: IPT-G Mini (4 sessions) in the context of mental health service delivery. Control group: standardised care with perinatal depression education for safety concerns and referral.	Feasibility of mhGAP-IG/IPT implementation in mental healthcare centres using mixed methods and multiple sources. Feasibility of the mhGAP/IPT-G mental health service delivery model: fidelity; engagement, quality, service perceptions. Feasibility of applying a trial design for mhGAP/ IPT-G impact evaluation: % exposed to intervention content, % data collection.	N/A
**Mpango (2017) (82)**	Uganda	Trial Registration: Cluster randomised controlled trial	Patients aged 18+ years living with HIV and attending HIV care at 40 public healthcare facilities in 3 study districts.	Intervention group: psychoeducation, Healthy Activity Program, antidepressants, referral to a specialist mental health worker. Control group: enhanced usual care including screening results, mhGAP-IG guidelines given to attending clinicians and referral to specialist mental health services.	Primary outcomes (6 months' follow-up): depression symptoms (PHQ-9), lack of remission (PHQ-9 >4). Secondary outcomes: (at 6 and 12 months): virological failure on ART, past 3 days' missed medication, mean CD4 count, WHO stage 3 or 4 deaths, quality adjusted life years (SF-6D), days out of work, functioning (WHODAS 2.0)	N/A
**Nakku et al. (2019) (83)**	Uganda	Uncontrolled study	Adult patients of 12 PHC clinics and 1 district hospital PHC department	PHC staff received 5 days' mhGAP-IG training, including identification, diagnosis and treatment. Staff were supervised by a specialist mental health worker.	Depression screening (PHQ-9), alcohol use disorder screening (AUDIT), epilepsy symptom (seizures in the last 30 days), functional impairment (WHODAS 2.0).	Clinical detection was increased at 3 but not 12 months' follow-up. Patients treated in PHC experienced significant reductions in symptom severity and functional impairment over 12 months. There was negligible change in population-level contact coverage for depression and alcohol use disorder.
**Smith et al. (2017) (84)**	Rwanda	Protocol: Uncontrolled study	Adults presenting to mental health clinics at 4 health centres with a diagnosis of major mental or neurological disorder.	Staff received 5 days' mhGAP-IG-based training followed by regular supervision, focused on schizophrenia, bipolar affective disorder, depression and epilepsy.	Primary outcomes: symptoms and functioning at baseline, 8 weeks and 6 months' follow-up using clinician-administered scales (GHQ-12 and WHODAS 2.0 Brief). Process evaluation: (1) routine service utilisation and supervision checklist data, (2) qualitative semi-structured interviews with PHC nurses, service users and family members.	- N/A
**Smith et al. (2020) (85)**	Rwanda	Uncontrolled study	2239 mental health service users at 19 PHC centres + Butaro district hospital + 1500 CHWs.	38 primary care nurses received 40 hours' training based on guidelines including mhGAP-IG. They received brief refresher courses every 6 months, supervision, clinical checklists to track care provision and systems-based quality improvement.	Attendance for mental healthcare at all centres during 2 year scale-up, implementation fidelity (clinical observation checklists during supervisory and quality improvement visits), symptoms (GHQ-12), disability (Brief WHODAS 2.0), economic burden.	Health centres achieved 70% and 76% of supervisory visits and clinical checklist utilisation target goals, respectively. GHQ-12 and WHODAS scores increased significantly at 6 months. Service users unable to work decreased and the proportion of caregivers leaving income-generating work decreased significantly.

Authors	Country	Study design	Sample	Intervention details	Evaluation details, if any	Summary of findings
**Dos Santos et al. (2019) (86)**	Mozambique	Uncontrolled study	Over 4 years:<br>Trained: 177 health professionals + 1161 CHWs<br>Diagnosed 13563 new cases<br>Awareness raised: >14,000 people per year.	Government advocacy, translation and adaptation of mhGAP epilepsy training materials, training and supervision of non-specialist health professionals and CHWs, population awareness raising and community involvement.	N/A	67% increase in epilepsy consultations, reduction of the epilepsy treatment gap from 99 to 96%. Early results led to increased purchasing of antiepileptic medication by the Ministry of Health.
**Madhombiro et al. (2019) (87)**	Zimbabwe	Randomised controlled trial	40 participants attending a tertiary HIV clinic (20 per arm) aged 18+ years and diagnosed with alcohol use disorder.	Intervention group: nurses received training in good clinical practice and ethical principles plus MI/CBT training.<br>Control group: nurses were trained in good clinical practice, ethical principles and mhGAP-IG for alcohol use.	Primary outcomes: (at baseline and follow-up): problematic alcohol use (AUDIT), viral load and CD4 (blood tests), disability (WHODAS 2.0), quality of life (WHOQoL).	There was a statistically significant decrease in AUDIT scores over time in both groups. At follow-up, median CD4 counts for intervention group and control groups showed improved immunity in both arms.
**Fairall et al. (2018) (88)**	South Africa	Protocol: Pragmatic cluster randomised controlled trial	Target size:<br>2000 patients on antiretroviral treatment screening positive for depression (PHQ-9) from 40 clinics across 2 rural districts.	PHC nurses in both arms were trained to use the mhGAP-IG.<br>Intervention group: communication skills training + 4 extra sessions on depression diagnosis and management.<br>Control group: training in identification and management of common chronic diseases, including mental health.<br>Nurses can refer patients diagnosed with depression for initial treatment to mental health specialists.	Primary outcomes: improvement in PHQ-9 at 6 months' follow-up, viral load suppression after 12 months.	- N/A
**Baron et al. (2018) (89)**	Ethiopia,<br>Uganda,<br>South Africa,<br>India,<br>Nepal	Protocol: Cohort study	17 treatment cohorts across 5 sites (total n=2182) including adults with depression and alcohol use disorder (n=200), adults with psychosis and epilepsy (n=150).	mhGAP-IG used to inform mental health care plans for detection and treatment of depression, alcohol use disorder, psychosis, epilepsy in routine PHC.	Primary outcomes: functioning (WHODAS 2.0), seizure frequency (epilepsy), AUDIT (alcohol use disorder), PHQ-9 score (depression).<br>Secondary outcomes: PHQ-9 (alcohol use disorder, psychosis and epilepsy).	N/A
**Hamdani et al. (2017) (90)**	Pakistan	Protocol: Hybrid cluster randomised controlled trial	540 parent-child dyads across 30 clusters (rural union councils)	Intervention: WHO Parents Skills Training delivered to caregivers of children with developmental disorders by 'family volunteers' using a tablet application.<br>Control: enhanced usual care (PHC physicians trained in mhGAP-IG developmental disorder module).	Primary outcome: child's functioning (WHODAS-Child) after 6 months.<br>Secondary outcomes: video-taped caregiver-child interaction, communication (CGI), emotional well-being (SDQ), health related quality of life (Peds-QL), family empowerment (FES); stigma (ISE), life events (LEDS).	N/A

Authors	Country	Study design	Sample	Intervention details	Evaluation details, if any	Summary of findings
**Robinson et al. (2019) (91)**	Pakistan	Trial registration: Cluster randomised controlled trial	Target size: 526 (263 each arm) participants aged 17-65 years diagnosed with schizophrenia or schizoaffective disorder in the catchment area of one of 24 PHC centres	Intervention group: mhGAP-IG training for PHC physicians (including psychiatrist liaison), regular, reliable provision of psychotropic drugs at PHC centres + family member training to dispense and administer medication + automated text message reminders to medication supervisors. Control group: enhanced usual care: mhGAP-IG training for PHC physicians (including psychiatrist liaison) + regular, reliable provision of psychotropic drugs at PHC centres.	Primary outcome: (at baseline, 6 and 12 months): functioning (GAF). Secondary outcomes: (at baseline, 6 and 12 months): symptoms (BPRS); caregiver burden (Family Burden Scale), physical health (BMI, blood pressure, health records), perceived stigma (Stigma of Mental Illness), cost (CSRI), quality-adjusted life years (EuroQoL), antipsychotic side effects (GASS), drug use (DAST-10), illness severity, treatment response (CGI), depression (PHQ-9), suicidality (SBQ-R).	- N/A
**Maulik et al. (2017) (92)**	India	Uncontrolled study	People attending 2 PHC clinics serving 30 villages (n=5167).	Training for doctors on mhGAP-IG, interviewing skills, diagnosis and treatment + mobile platform algorithms to share clinical data between accredited social health activists + stigma reduction campaign.	Primary outcome: mental health service use post-intervention, depression and anxiety scores at follow-up among those scoring PHQ-9 or GAD-7 ≥10 at baseline, stigma, mental health awareness (knowledge, attitude and behaviour about mental health instrument, BACE-TS).	21 accredited social health activists and 2 PHC doctors were trained. 5007 of 5167 eligible individuals were screened and 238 referred to PHC doctors for management of a CMD, of whom 30 attended. Depression and anxiety scores improved significantly. Stigma and mental health awareness improved in the community.
**Elshazly et al. (2019) (93)**	Bangladesh	Descriptive account	PHC staff (e.g. GPs).	Recruitment of psychiatrists full or part-time to train PHC staff to assess and manage priority MNS disorders using mhGAP-IG.	N/A	Challenges: inconsistent psychotropic medication availability, difficulty retaining local psychiatrists, providing supervision.
**Tarannum et al. (2019) (94)**	Bangladesh	Descriptive account	62 PHC staff doctors (n=36), medical assistants (n=10), health educators (n=7), psychologists (n=6), medical coordinators (n=2) and nurses (n=1).	2x 3 day mhGAP-HIG training workshops were delivered by psychiatrists and a psychologist to staff working with Rohingya refugees. 15 trained staff participated in weekly supervision and at-work training visits.	N/A	Almost 1,200 mental health consultations were conducted, an increase from 40 to 160 per month. Supervision highlighted the need to improve skills of mental state examination, psychoeducation and psychosocial support.
**Jha & Sapkota (2013) (95)**	Nepal	Descriptive account	Psychiatrists and neurologist in hospital or clinic settings	mhGAP-IG-based dementia assessment and management protocol for doctors and post-diagnostic education and training protocol for people with dementia and their caregivers.	N/A	A clinic-based diagnostic procedure was developed in parallel with mhGAP-IG-based dementia training and education for caregivers, adapted for the Nepalese context.
**Jordans et al. (2017) (96)**	Nepal	Uncontrolled study	509 community informants, identified using a detection tool	Community informants were trained to use a case-finding tool. They used the tool to identify people with mental disorders, whom they referred to facilities with mhGAP-IG-trained staff.	Case finding, people accessing care, people initiating treatment.	67% of identified cases (n=341) accessed mental healthcare and 77% (n=264) of them initiated treatment. People in the rural district (Pyuthan) were more likely to access healthcare than those living in Chitwan district.

Authors	Country	Study design	Sample	Intervention details	Evaluation details, if any	Summary of findings
**Breuer et al. (2018) (97)**	Nepal	Case study	10 facilities where mental healthcare plans (MHCPs) implemented (including mhGAP guidelines)	Facility MHCPs plans included staff awareness, screening and assessment following mhGAP-IG, brief psychosocial support from the Healthy Activity Programme and Counselling for Alcohol Problems, medication and follow-up monitoring.	Quarterly collection of facility-level indicators: medication supply, facility and community supervision, human resources, psychosocial interventions including mhGAP, referrals to tertiary care, community identification and awareness.	Conditions for high mental health service utilisation: basic and advanced psychosocial care provision, mhGAP-IG, referral to tertiary services, trained female community health volunteers, medication supply, trained staff, a community informant detection tool or greater attendance at community awareness activities.
**Jordans et al. (2019) (98)**	Nepal	Uncontrolled study	Community surveys (n=3,482), PHC clinics (n=3,627), treatment of depression (n=449), alcohol use disorder (n=137), psychosis (n=95) and epilepsy (n=42).	Facility-level packages included training and supervision for health workers to detect, diagnose and initiate treatment for depression, psychosis, alcohol use disorders and epilepsy, following the mhGAP-IG. Community-level packages included community sensitisation and case-finding. Service packages included medication provision.	Contact coverage, system contact, depression (PHQ-9), alcohol use (AUDIT, Drinker Inventory of Consequences), psychosis (PANSS), past-month seizure frequency, functioning (WHODAS 2.0), diagnostic accuracy, minimally adequate treatment for depression and alcohol use disorder (mhGAP-IG).	Contact coverage increased by 7.5% (alcohol use disorder) 12% (depression), 12% (epilepsy), and 50% (psychosis). Depression detection increased by 10% 24 months post-training. Alcohol use disorder detection increased by 11% at 24 months. Minimally adequate treatment was 67% for depression and 75%, for alcohol use disorder at 24 months. There was a 6.4-point reduction in psychosis and 7.2-point reduction in depression, 12 months post-treatment.
**Jordans et al. (2019) (99)**	Nepal	2 pragmatic randomised controlled trials	120 participants with depression (intervention: n=60, control: n=60) + 162 participants alcohol use disorder (intervention: n=82, control: n=80) across 10 PHC facilities in Chitwan	<u>Intervention</u>: mhGAP-IG training for PHC staff and individual psychological treatments (Healthy Activity Program (HAP) for depression and Counselling for Alcohol Problems (CAP)).<br>Both arms had psychoeducation and access to psychotropic medications.	<u>Primary outcomes</u>: symptom severity 12 months post-enrolment: depression (PHQ-9), alcohol use disorder (AUDIT), functional impairment (WHODAS).	Participants with depression in the intervention arm had greater reductions in PHQ-9 and WHODAS scores compared to participants in the control arm.<br>No significant effect was found when comparing control and intervention participants for alcohol use disorder.
**Jordans et al. (2020) (100)**	Nepal	Non-randomised controlled trial	2044 participants attending one of 10 facilities where MHCPs were implemented	<u>Intervention group 1</u>: PHC staff trained to detect, diagnose and begin treatment following the mhGAP-IG<br><u>Intervention group 2</u>: mhGAP-IG-trained PHC + psychological treatment: HAP (depression), CAP (alcohol).<br>Control: usual care.	<u>Primary outcomes</u>: depression (PHQ-9), alcohol (AUDIT), disability (WHODAS).<br><u>Other outcomes</u>: perceived quality of patient centred care for chronic illness (PACIC), patient-rated staff empathy, therapeutic alliance, psychoeducation, communication, other skills (ENACT).	Depression symptoms and disability improved significantly in intervention group 2, relative to controls. Alcohol use disorder and disability improved significantly in both intervention groups relative to controls. Patient-rated staff skills were associated with clinical improvement of depression but not alcohol use disorder.
**Upadhaya et al. (2020) (101)**	Nepal	Non-randomised controlled trial	10 intervention healthcare facilities and 10 control healthcare facilities in Chitwan	<u>Intervention</u>: health facilities implementing MHCPs for depression, epilepsy, psychosis and alcohol use disorders. Packages included 9 days' mhGAP-IG-based training for PHC staff.<br><u>Control</u>: usual physical health services	Assessment of Chronic Illness Care.	At follow-up, 90% of intervention facilities had basic support for chronic illness care and 10% had full support, compared with 10% at baseline. At follow-up, 20% of control health facilities had limited support for chronic illness care and 80% had basic support.
**Luitel (2020) (102)**	Nepal	Uncontrolled study	A random sample of adults with depression and alcohol use disorder in Chitwan district (n=1983 at baseline and n=1499 at follow-up)	Faculty-level package of the PRIME project included training and supervision of health workers to detect, diagnose, treat and refer MNS disorders according to the mhGAP-IG.	Contact coverage for participants with a past-year depressive episode, scoring PHQ-9 >9 or AUDIT >8, depression (PHQ-9) and alcohol use disorder (AUDIT) screening, barriers to seeking mental healthcare (BACE).	12% (n=13) of people with depression and 10% (n=9) of people with alcohol use disorder had received mental health treatment in the past 12 months – not significantly different to baseline. Overall BACE scores improved significantly.

**Authors**	**Country**	**Study design**	**Sample**	**Intervention details**	**Evaluation details, if any**	**Summary of findings**
**Luitel et al. (2020) (103)**	Nepal	Process evaluation	First workshop: District-level stakeholders (n=14) and policymakers (n=10)<br>Last two workshops: district-level stakeholders, (n=11) and policymakers (n=8)	mhGAP-IG-based district MHCPs developed for the PRIME project. At health workers were trained to detect, diagnose and treat mental disorders using the mhGAP-IG, under supervision.	Implementation process indicators, e.g. contextual factors affecting implementation, availability of resources, reach and coverage of services, training and supervision, staff, patient and caregiver perspectives on service acceptability and feasibility, barriers and facilitators to MHCP implementation.	Out of 32 indicators, 21 (66%) were fully achieved, 10 (31%) partially achieved and 1 (3%) not achieved. The proportion of PHC patients receiving mental health services increased by 1200% over the 3 year implementation period.<br>Barriers: staff turnover, lack of confidential space for consultation, no mental health supervision in the existing system, stigma.<br>Facilitators: Ministry of Health involvement, psychotropic medicine procurement through PRIME, health worker motivation, supervision.
**Aldridge et al. (2020) (104)**	Nepal	Randomised control trial	Screening: 2044 people attending 10 health centres<br>Depression study: Intervention group (n=173), control group (n=72).<br>Alcohol study: intervention group (n=174), control group (n=57).	Intervention group: psychotropic medication, psychosocial support or psychoeducation according to the mhGAP-IG<br>Control group: HAP (depression) or CAP (alcohol use disorders).	Primary outcome: recent suicidal ideation (responses to text questionnaires on electronic tablet), depression (PHQ-9), alcohol use (AUDIT).	Suicidal ideation reduced significantly for depressed participants in the intervention group relative to the control groups at 3 and 12 months' follow-up.<br>Suicidal ideation among people with alcohol use disorders was not significantly different between intervention and control groups at 3 or 12 months' follow-up.
**Kohrt et al. (2020) (105)**	Nepal, Nigeria	Trial registration: cluster randomised controlled trial	Target size: 3300 government or clinic-employed PHC staff with mhGAP-relevant roles and responsibilities.	Intervention group: training and supervision of PHC workers using a digital mhGAP-IG with patients.<br>Control group: training and supervision of PHC workers using the paper-based mhGAP-IG with patients	Primary outcome: documentation of depression in clinical records (mhGAP depression diagnosis tool) by PHC staff<br>Secondary outcomes: depression (PHQ-9), disability (WHODAS), staff knowledge and skills including patient-reported (ENACT), stigma (Social Distance Scale), acceptability, feasibility, organisational readiness change, normalisation, supervisory support, attitudes towards depression.	N/A
**Anjara et al. (2019) (106)**	Indonesia	Pragmatic (partially) randomised cluster non-inferiority trial	Patients screening positive for psychiatric morbidity: Intervention arm (n=174) + Control arm (n=151) across 14 intervention and 14 control clinics	Intervention: GPs and nurses selected for mhGAP-IG-based training, after which they delivered pharmacological and/or psychosocial therapy according to mhGAP-IG and/or refer to specialist.<br>Control: clinical psychologist intervention (combination of basic and advanced psychosocial therapies).	Primary outcome: health and social functioning (HoNOS).<br>Secondary outcomes: disability (WHODAS 2.0), health-related quality of life (EQ-5D-3L), demographic information and health service utility (CSRI), psychiatric morbidity (CIS-R), costs.	The intervention arm was statistically not inferior to the control arm in improving social and physical impairment, disability and health-related quality of life at 6 months.<br>Economic analyses found lower costs and better outcomes in the control arm, suggesting a 50% probability of the mhGAP framework being cost-effective at the Indonesian threshold per QALY.
**Powell & Brack (2019) (107)**	Philippines, Guinea	Descriptive account	Non-mental health personnel (Philippines)	Philippines: mhGAP-IG-based training to meet needs post-typhoon Haiyan in 2013 (Philippines).<br>Guinea: mhGAP-IG-based training to support Ebola survivors.	N/A	N/A

Keynejad R, *et al. Evid Based Ment Health* 2021; 24:124–130. doi: 10.1136/ebmental-2021-300254

Authors	Country	Study design	Sample	Intervention details	Evaluation details, if any	Summary of findings
**Budosan et al. (2020) (108)**	Philippines<br>Haiti	Descriptive account	Philippines: 130 medical doctors + 160 public health nurses trained in mental health + 609 health workers training in psychosocial support.<br><br>Haiti: 115 healthcare providers + 190 community psychosocial workers.	Philippines: psychosocial support training post-Typhoon Haiyan, followed by at-work training sessions using mhGAP protocols and guidelines.<br>Haiti: a community-based integrated mental health and psychosocial support programme in earthquake-affected and displaced population areas. 3 cycles (90 hours) of theoretical mental health training were delivered to health workers in 5 departments and community psychosocial workers in 5 departments.	From document searches:<br>Community level: mental health service capacity, availability, access and affordability<br>Individual level: perceived stress, wellbeing and resilience, symptom severity and functional status	Philippines: 98% of PHC units in Eastern Visayas had at least 1 mhGAP-IG-trained health worker, ~800 patients received clinical interventions, psychotropic medication access improved, health workers did outreach and psychoeducation.<br>Haiti: mental health knowledge improved in CHWs by 46% and in PHC staff by 75%, access to mental healthcare increased in 5 departments, psychotropic medications became available. 616 patients received clinical interventions, 65,000 beneficiaries received psychosocial interventions in 5 departments, psychological interventions reduced distress by 48%, improved wellbeing by 27% and resilience by 31%.

Abbreviations: AUDIT: alcohol use disorders identification test, BACE-TS: barriers to access to care evaluation (treatment scale), BMP-P: brief problem monitor for parents, BPIRC: brief physical impairment rating checklist, BPRS-E: brief psychiatric rating scale-extended, BSS: Beck suicidal ideation scale, CAMI: community attitudes towards the mentally ill, CAP: counselling for alcohol problems, CBCL: child behaviour checklist, CBT: cognitive behavioural therapy, CFIR: consolidated framework for implementation research, CGI: clinical global impressions scale, CHW: community health worker, CIDI: composite international diagnostic review, CIS-R: clinical interview schedule-revised, CMD: common mental disorder, CPN: community psychiatric nurse, CSRI-PND: client service receipt inventory (postnatal depression), DAST-10: drug abuse screening test, DISC-12: discrimination and stigma scale, DRC: Democratic Republic of Congo, DSM: diagnostic and statistical manual, EDSS: electronic decision support system, ENACT: enhancing assessment of common therapeutic factors, EuroQol/EQ-5D: health quality questionnaire, FABI: fear and behavioural intentions, FBIS: family burden interview scale, FES: family empowerment scale, FH: faith healer, GAF: global assessment of functioning, GASS: Glasgow antipsychotic side effects scale, GHQ-12: general health questionnaire, GP: general practitioner, HFIAS: household food insecurity access scale, HIV: human immunodeficiency virus, HOME-IT: home observation for the measurement of the environment inventory, HoNOS: health of the nation outcome scales, IAT: implicit association test, ISE: inventory of stigmatising experiences, IPT-G: interpersonal psychotherapy-group, IPV: intimate partner violence, ISMI: internalised stigma of mental illness, JCQ: job content questionnaire, JSQ: job stress questionnaire, LEDS: life events and difficulties schedule, LIFE-RIFT: longitudinal interval follow-up evaluation range of impaired functioning tool, LTE: list of threatening experiences, MAMAS: maternal adjustment and maternal attitudes questionnaire, MAKS: mental health knowledge schedule, MCQ: multiple-choice question, MHCP: mental healthcare plan, mhGAP-HIG: mental health gap action programme (humanitarian) intervention guide, mHealth: mobile health, MHNCCS: mental health nursing clinical confidence scale, MI: motivational interviewing, mental illness clinicians attitudes, MINI: mini international neuropsychiatric inventory, MNS: mental, neurological and substance use, MSc: Master of Science, NCD: non-communicable diseases, N/A: not applicable, OPCRIT: operational criteria checklist for psychotic illness and affective illness, OSSS-3: Oslo social support scale, PACIC: patient assessment of chronic illness care, PAHO: Pan-American Health Organisation, PANSS: positive and negative syndrome scale, PCQ: parent concerns questionnaire, PedsQL: paediatric quality of life inventory, PHC: primary healthcare, PHQ-9: patient health questionnaire, PICSS: perinatal infant care social support, PSQ: patient satisfaction questionnaire, QALY: quality-adjusted life year, RCQ: readiness for change, RCT: randomised controlled trial, RIBS: reported and intended behaviour scale, SBQ-R: suicide behaviours questionnaire-revised, SDS: social distance scale, SDQ: strengths and difficulties questionnaire, SF-6D: short form six dimension health index, SIDAS: suicide ideation attributes scale, SEMI: short explanatory model interview, SMI: severe mental illness, SMS: short messaging service, TH: traditional healer, ToC: theory of change, WERCAP: Washington early recognition centre affectivity and psychosis, WHODAS: WHO disability assessment schedule, WHOQOL-BREF: brief WHO quality of life questionnaire, WPA: World Psychiatric Association, YSR: youth self-report.

Keynejad R, *et al. Evid Based Ment Health* 2021; 24:124–130. doi: 10.1136/ebmental-2021-300254

Table 6: Summary of included studies reporting uses of the mhGAP-IG in Research

Authors	Country	Study design	Sample	Study details	Outcome measures	Summary of findings
**Aiyenigba et al. (2019) (109)**	Nigeria	Descriptive account	Couples experiencing infertility	The Fertility Life Counselling Aid (FELICIA) is an adaptation of the Thinking Healthy Programme (THP), a Cognitive Behavioural Therapy (CBT)-based intervention for perinatal depression. FELICIA counsellors are familiarised with the mhGAP-IG to identify severe depression and anxiety.	A pilot RCT is taking place in Nigeria to assess feasibility prior to a full RCT.	N/A
**Geibel et al. (2016) (110)**	Ethiopia	Validation study	134 15-18 year-old participants (50% female), vulnerable to HIV infection	Trained nurses administered a translated, adapted version of the Youth Self Report (YSR) screening tool before participants were assessed by a psychiatrist. The YSR was repeated one month later.<br>Study participants receiving a psychiatric diagnosis were referred for treatment in accordance with the mhGAP-IG.	Sensitivity and specificity of YSR compared to psychiatrist assessment were calculated to assess validity.	The adapted, personally-administered Amharic YSR had sufficient reliability and validity to identify young women with anxiety or depression and/or social problems and young men with attention problems.
**Ambaw et al. (2015) (111)**	Ethiopia	Protocol: prospective cohort study	703 patients with a new diagnosis of tuberculosis (469 without depression and 234 with depression)	N/A	N/A	The study will explore whether receipt of mhGAP-IG-recommended treatment for depression moderates anti-tuberculosis treatment outcomes.
**Asher et al. (2016) (112)**	Ethiopia	Protocol: cluster randomised controlled trial	182 people diagnosed with schizophrenia receiving treatment in primary care, from an existing cohort, across 54 sub-districts	<u>Intervention arm</u>: Manualised community-based rehabilitation by trained and supervised staff, plus EUC.<br><u>Enhanced usual care (EUC) arm</u>: Facility-based care only: mhGAP-IG recommended treatment from a nurse or health officer in primary care (antipsychotic medication, basic psychoeducation and follow-up), plus referral to a psychiatric nurse outpatient clinic or psychiatric hospital if needed.	N/A	N/A
**Keynejad et al. (2020) (113)**	Ethiopia	Protocol: Randomised controlled feasibility trial	Target size:<br>75 pregnant women attending antenatal care, scoring 5+ on PHQ-9 and functional impairment	All staff have been trained, through the PRIME project, on the mhGAP-IG.<br><u>Intervention group 1</u>: 4 sessions of PST-IPV<br><u>Intervention group 2</u>: 4 sessions of standard PST<br><u>Control group</u>: information about sources of support (enhanced usual care)	<u>Primary outcomes:</u> (baseline, 9 weeks): depression (PHQ-9).<br><u>Secondary outcomes:</u> (baseline, 9 weeks): post-traumatic stress (PCL-5), anxiety (GAD-7), disability (WHODAS 2.0), past-month IPV, self-efficacy, mastery, social support (OSSS-3), healthcare use (CSRI), obstetric and neonatal outcomes, gender attitudes.	- N/A

Authors	Country	Study design	Sample	Study details	Outcome measures	Summary of findings
**Smith Fawzi et al. (2020) (114)**	Tanzania	Protocol: cluster randomised controlled trial	742 pregnant women (up to 30 weeks' gestation) aged 18 years or over, HIV positive, receiving antiretroviral treatment, scoring 9+ on PHQ-9, across 16 clusters	Intervention arm: 1 orientation session plus 6 sessions of the Healthy Options group psychosocial support intervention, comprising components of problem solving therapy and CBT. The intervention was adapted from the Thinking Healthy Programme and was delivered in Swahili by lay community-based health workers (CBHW).<br>EUC arm: 1 day training session for clinic staff using mhGAP-IG, on screening, assessment and management of depression, including referral for specialist care, such as antidepressant medication, counselling and support for suicidal ideation.	Primary outcome: PHQ-9 score at 6 weeks and 9 months postpartum.	N/A.
**Wainberg et al. (2018) (115)**	Mozambiq ue	Protocol: cluster randomised controlled trial	901 clinical staff aged 18-65 years	Arm 1: Specialty mental healthcare: mhGAP-IG content (psychiatric medications and evidence-based psychotherapies) provided by existing specialists.<br>Arm 2: Integrated care: mhGAP-IG content (psychiatric medications and evidence based psychotherapies) provided by primary care staff<br>Arm 3: Clinic stepped care: mhGAP-IG content (psychiatric medications provided by primary care staff and evidence-based psychotherapies provided by community health workers).	Primary outcome: Reach and costs of each arm.<br><br>Secondary outcome: Retention and Clinical Symptoms at 12 months' follow-up for each arm.	.N/A
**Odenwald et al. (2012) (116)**	Somaliland	Non-randomised controlled trial	(a) 17 outpatients experiencing acute psychosis and<br>(b) 18 patients with chronic psychotic disorders in remission.<br>Both groups were using Khat.	Phase 1: (a) Intensive 6 week home-based care package.<br><br>Phase 2: Both groups then received community-based relapse prevention, comprising mhGAP recommendations of psychoeducation, low-dose neuroleptic treatment, monthly home visits and counselling.	Measured Brief Psychiatric Rating Scale (BPRS) scores three times, functioning, khat use and other outcomes, with 10 month follow-up.	Group (a) improved significantly after home-based care and did not differ significantly from group (b) at the start of Phase 2. After Phase 2, group (a) had higher BPRS positive and negative symptom scores than group (b). All patients' BPRS scores worsened with time, except for depressive symptoms.<br>Levels of functioning increased in 20/33 participants. Most experienced improvements in basic functioning, such as communication and self-care.<br>Khat use reduced in group (b).
**Khan et al. (2017) (117, 118)**	Pakistan	Randomised controlled trial	71 pregnant women identified by a prior study scoring 9+ on the Self-Reporting Questionnaire (SRQ)	Intervention arm (n=34): 2x psychoeducation sessions ('Happy Mother, Healthy Child in Ten Steps') informed by the mhGAP-IG, delivered at home by local community health workers.<br>Control arm (n=37): routine antenatal care.	Primary outcome: help-seeking for psychological distress, measured by a semi-structured interview at 2 months post-intervention.	More women in the intervention arm sought assistance for distress from community health workers than in the control arm.

Authors	Country	Study design	Sample	Study details	Outcome measures	Summary of findings
**Sikander et al. (2019) (119)**	Pakistan	Cluster randomised controlled trial	570 pregnant women with moderate or severe symptoms of depression (PHQ-9 of 10+) across 40 village clusters	Intervention arm: 18 sessions of the Thinking Healthy Program Peer-delivered plus (THPP+) group-based psychosocial intervention plus EUC. Six monthly sessions were followed by 2-monthly sessions until 36 months post-partum. The intervention included peer support, behavioural activation and problem solving, with developmental activities for children.<br>EUC arm: Informing participants about their depression diagnosis, ways to seek help for it (including an information sheet), informing their health worker and providing primary care facility-based physicians with mhGAP guidelines adapted for perinatal depression.	Follow-up at 3 and 6 months post-partum to measure depression symptoms and remission (PHQ-9 score <10).	At 6 months' follow-up, there were no significant differences in PHQ-9 scores and proportions with remission between the intervention and EUC arms.
**Maselko et al. (2020) (120)**	Pakistan	Cluster randomised controlled trial	As above	As above	Follow-up at 3, 6, 12, 24 and 36 months post-partum as above and to measure child socioemotional skills (SDQ-TD).	Intervention and EUC groups showed high remission rates and reduced symptom severity at 36 months' follow-up. There were no statistically significant differences between child outcomes in the intervention and EUC groups.
**Surkan et al. (2020) (121)**	Pakistan	Protocol: randomised controlled trial	Approximately 1200 pregnant women up to 22 weeks' gestation	Intervention arm: 6 sessions of the individual Happy Mother, Happy Baby (HMHB) intervention (plus 2-6 booster sessions), based on CBT and relaxation techniques, delivered by non-specialist providers, to address anxiety symptoms during pregnancy. The intervention draws on core principles and strategies of the Thinking Healthy Programme.<br>EUC arm: access to the recommended 8 antenatal visits evaluating health, discussion concerns and performing routine examinations. Hospital staff will be trained in the depression module of the mhGAP-IG.	Primary outcome: diagnosis of postpartum common mental disorders using the SCID at 6 weeks postpartum.	N/A
**Nadkarni et al. (2015) (122)**	India	Feasibility study	53 men scoring 12+ on the AUDIT alcohol use questionnaire	Intervention arm: 3 phases comprising 1-4 sessions of Counselling for Alcohol Problems (CAP), incorporating motivational interviewing and problem solving components.<br>EUC arm: Informing the patient's primary care physician of their AUDIT score and providing them with the mhGAP-IG guidelines for substance use disorders.	Acceptability and feasibility of CAP was assessed using a clinical case series, with CAP delivered by 4 mental health professionals and 19 lay counsellors.	13 participants completed treatment and the remainder dropped out. There were non-significant reductions in past 2 weeks' alcohol consumption, mean AUDIT score and alcohol-related problems in the CAP arm Relative to the EUC arm.

Authors	Country	Study design	Sample	Study details	Outcome measures	Summary of findings
**Nadkarni et al. (2017) (123)**	India	Randomised controlled trial	377 men scoring 12-19 on the AUDIT alcohol use questionnaire (harmful use)	As above	Primary outcomes were remission (AUDIT score <8) and past 2 weeks' daily alcohol consumption at 3 months' follow-up.	Significantly higher proportions of participants in the CAP + EUC arm showed remission and abstinence at 3 months' follow up than in the EUC arm but there was no significant difference in mean past 2 weeks' daily alcohol consumption among those who had been drinking in that period.
**Nadkarni et al. (2017) (124)**	India	Randomised controlled trial	As above	As above	As above, at 12 months' follow-up.	Significantly higher proportions of participants in the CAP + EUC arm showed remission and abstinence at 12 months' follow up than in the EUC arm. The intervention effect for remission was higher at 12 months than at 3 months.
**Patel et al. (2017) (125)**	India	Randomised controlled trial	495 individuals aged 18-65 years with moderately severe to severe depression (>14 on PHQ-9)	Intervention arm: 6-8 sessions of the Healthy Activity Program (HAP) behavioural activation intervention, delivered by lay counsellors, plus EUC.<br>EUC arm: screening results were provided to the patient and primary care physician. Physicians were provided with an mhGAP-IG focused on depression and information about referrals to psychiatric care.	Primary outcomes were severity of depression symptoms on the BDI-II and remission from depression (PHQ-9 <10) at 3 months' follow-up.	Participants in the intervention arm had significantly less severe depressive symptoms and higher remission than the EUC arm at follow-up. Secondary outcomes of disability, days out of work, intimate partner violence victimisation (women), behavioural activation and suicidal thoughts or attempts were also significantly improved.
**Weobong et al. (2017) (126)**	India	Randomised controlled trial	As above	As above	As above, at 12 months' follow-up.	Improved depressive symptoms and remission at 3 months' follow-up in the intervention arm maintained at 12 months. No difference in secondary outcomes at 12 months.
**Prabhakaran et al. (2019) (127)**	India	Randomised controlled trial	3698 participants with hypertension and diabetes across 40 community health centre clusters	Intervention arm: mWellcare: electronic health records and decision support for integrated management of hypertension, diabetes, tobacco and alcohol use and depression (using mhGAP-IG).<br>EUC arm: training of physicians and nurses on clinical management guidelines for hypertension and diabetes, management charts displayed prominently in outpatient clinics, lifestyle advice pamphlets in local languages explained to each participant.	Primary outcomes: systolic blood pressure and glycated haemoglobin at 12 months' follow-up.<br>Depression (PHQ-9) was only measured at follow-up and alcohol consumption (AUDIT) only in the intervention arm.	There was no evidence that mWellcare improved health outcomes beyond the effects of EUC.

Authors	Country	Study design	Sample	Study details	Outcome measures	Summary of findings
**Spector et al. (2019) (128)**	India<br>Brazil<br>Tanzania	Protocol: implementation study	150 participants with dementia across 3 countries, identified using mhGAP-IG for Phase 3.	Phase 1: exploring barriers to implementation of Cognitive Stimulation Therapy (CST) in each country via meetings with clinician, policymaker, patient and family stakeholders.<br>Phase 2: developing implementation plans for each country.<br>Phase 3: evaluating implementation plans through studying CST in each country (n=50, total n=150).<br>Phase 4: refining and disseminating implementation strategies.	Adherence to CST, attendance, acceptability, cost and attrition and agreed clinical outcomes such as cognition, quality of life, activities of daily living.	N/A
**Kohrt et al. (2020) (129)**	Nepal	Protocol: cluster randomised controlled trial	1750 participants across 24 municipality clusters	<u>Intervention arm:</u> 7 day mhGAP-IG-based training course plus recovery testimonials by mental health service user co-facilitators and testimonies and myth-busting sessions by aspirational figures.<br><u>Treatment as usual:</u> 7 day mhGAP-IG-based training course.	Primary outcomes: social distance scale at 6 months post-training and SCID-5 accuracy of clinical diagnoses at 3 months post-patient enrolment.	N/A
**Sangraula et al. (2020) (130, 131)**	Nepal	Feasibility study	121 adults (83% female) scoring >2 on the GHQ and >16 on WHODAS 2.0.	<u>Intervention arm:</u> 5 sessions of 2.5-3 hour group Problem Management Plus (PM+), comprising problem solving, stress management, behavioural activation and accessing social support. PM+ was delivered by trained, supervised gender-matched non-specialist community psychosocial workers.<br><u>Control arm:</u> EUC: brief psychoeducation and option for referral to PHC staff trained in mhGAP-IG management.	Primary clinical outcome: PHQ-9 depression symptoms at 8-8.5 weeks post-baseline.<br>Feasibility: fidelity and retention of participants, acceptability: in-depth interviews with participants, family members, programme staff and other stakeholders.	Average attendance: 4/5 sessions.<br>Group PM+ was acceptable and feasible.<br>Although not powered to assess effectiveness, the primary clinical outcome was larger in the intervention than the control arm.
**Van't Hof et al. (2020) (132)**	Nepal	Protocol: cluster randomised controlled trial	576 people aged 18 years and over, affirming the heart-mind screener and functional impairment (WHODAS >15), living in one of 72 district clusters.	<u>Intervention arm:</u> 5 sessions of 2.5-3 hour locally adapted group PM+.<br><u>Control arm:</u> 1x 30-45 minute family meeting with local community informant providing basic information on adversity and mental health, benefits of getting support and information about accessing services from mhGAP-trained staff.	Primary outcome: individual psychological distress (GHQ-12) at 12+1-2 weeks post-final session for intervention arm participants and 20+/- 1 weeks post-baseline for EUC participants	N/A
**Li et al. (2018) (133)**	China	Cluster randomised controlled trial	327 primary school-educated participants aged 18-50 years, diagnosed with schizophrenia, taking antipsychotic medication, across 2 clusters.	<u>Intervention arm (n=169)</u>: a manualised intervention comprising strategies against stigma and discrimination, psycho-education, social skills training and CBT, informed by the mhGAP-IG.<br><u>Control arm (n=158):</u> a face-to-face interview with a community psychiatrist.	Primary outcomes were ISMI, DISC-12, GAF, SQLS, SES, BPRS and PANSS-N scores at 6 and 9 months' follow-up.	Intervention arm participants showed reduced anticipated discrimination, BPRS and PANSS-N negative symptom scores, and increased DISC-12 overcoming stigma and GAF scores at 9 months' follow-up, relative to the control arm.

Authors	Country	Study design	Sample	Study details	Outcome measures	Summary of findings
**Tay et al. (2020) (134)**	Malaysia	Randomised controlled trial	331 refugees from Myanmar aged 18+ years with PSTD, complex PTSD, generalised anxiety disorder, depression or persistent complex bereavement.	Intervention arm 1 (n=170): 6x 45 minute weekly sessions of Integrative Adapt Therapy (IAT). Intervention arm 2 (n=161): 6x 45 minute weekly sessions of CBT. The mhGAP-IG was used to exclude prospective participants with symptoms of psychosis and a modified mhGAP-HIG suicide module was used to assess suicide risk.	Primary outcomes: PTSD, complex PTSD, major depressive disorder, Adaptive Stress Index (ASI) and Connor-Davidson Resilience Scale scores at 6 weeks post-treatment.	Intention-to-treat analysis showed greater reductions of all common mental disorder symptoms in the IAT arm relative to the CBT arm, most ASI domains and increases in resilience scores.
**Alhuwail et al. (2020) (135)**	Arabic-speaking countries	Scale development	N/A	The authors reviewed charge-free anxiety and depression apps available to Arabic speakers. They categorised the apps found on the Google Play store and Apple iOS store by their function using a system inspired by the mhGAP-IG,	N/A	23 apps were identified, with most (n=16) providing general information about anxiety, depression or both. 6 apps were spiritual or religious (5 Islamic and 1 Christian) and 5 apps focused on alternative treatments.

Table 7: Summary of included studies describing country-specific mhGAP-IG adaptation or contextualisation

Authors	Country	Study design	Sample	Summary of findings
**Gómez-Carrillo et al.(2020) (136)**	N/A	Theoretical	N/A	The authors developed a framework propose three domains to integrate cultural knowledge, structural competence and ethics into mhGAP planning, adaptation, training and implementation:<br>(1) Concepts of wellness and illness: examining cultural norms, knowledge, values and attitudes in relation to 'the culture of the mhGAP'.<br>(2) Systems of care: recognising formal and informal systems of care in the cultural context of practice.<br>(3) Ethical space: considering power dynamics, communication and decision-making.
**Faregh et al. (2019) (137)**	Haiti<br>Chad<br>Guinea<br>Nigeria<br>Ethiopia	Qualitative study	5 authors	The authors reflected on their experience of mhGAP-related projects in 6 countries. They identified 6 contextual issues requiring consideration during mhGAP implementation:<br>(i) Cultural differences in attitudes towards mental disorders and explanations thereof.<br>(ii) The local healthcare system's structure.<br>(iii) The degree of supervision and support that is available after training.<br>(iv) Trainee's prior education, knowledge and skills.<br>(v) Trainee recruitment processes.<br>(vi) The region's larger socio-political context.<br>They identified three approaches to address these issues:<br>(1) Cultural and contextual adaptation to training activities.<br>(2) Meaningful stakeholder and community engagement and<br>(3) Processes that support trainees, such as continuing supervision and communities of practice.
**Eaton et al. (2018) (138)**	Nigeria	Descriptive account	16 partners, community mental health experts, implementation experts, Federal and State Ministries of Health, international facilitators.	The authors conducted:<br>(1) Literature review of relevant evidence for appropriate and effective services.<br>(2) Situational analysis of available information on local mental healthcare.<br>(3) Theory of Change (ToC) workshop and generation of a ToC map.<br><br>These informed plans for PHC workers (nurses, community health officers, community health extension workers) at 2 sites to be trained to deliver a basic package of mental healthcare based on the mhGAP-IG. Master trainers will be trained and will then train local mental health leaders, who will be responsible for initial (base) training and regular refresher training.
**Spagnolo et al. (2019) (139, 140)**	Tunisia	Descriptive account	N/A	The authors describe the process of adapting the mhGAP-IG for the Tunisian setting by identifying mental health needs in greater Tunis in discussion with Ministry of Health officials, consulting epidemiological studies, preparing a preliminary training plan and conducting field observations in primary care clinics. The authors describe selecting training modules, tailoring the training programme to suit health workers' working hours, linguistic considerations, supervision decisions, adaptations for local mental health priorities and adjustments to treatment recommendations according to their local availability.
**Fekadu et al. (2016) (141)**	Ethiopia	Qualitative study	Key stakeholder groups in Sodo District.	Mental healthcare plans were developed and guided by mhGAP-IG guidelines, informed by in-depth interviews and focus group discussions. . Feasibility and acceptability of integrating mental health into primary care were evaluated via:<br>Situational analysis: cross-sectional data on health and factors likely to influence population health.<br>Asset mapping: community resource inventory.<br>Theory of Change workshops: to determine intervention outcomes and pathways required for these outcomes. Packages planned pertained to community (case detection, reintegration and inclusion), facility (capacity building, decision support and staff wellbeing) and organisational (programme management, supervision and sustainability) levels.

Authors	Country	Study design	Sample	Summary of findings
**Bitta et al. (2019) (142)**	Kenya	Qualitative study	29 nurses<br>3 public health officers<br>28 traditional health practitioners (THPs)	Focus group discussions identified local terms for depression, psychosis, epilepsy, substance use disorders, self-harm and suicide, but not dementia. Child and adolescent mental health problems were considered consequences of poor parenting. Participants ascribed biological or supernatural causes to mental health problems. THPs were prepared to refer patients to health services but primary care staff were not prepared to collaborate with THPs because they perceived them to worsen outcomes.<br>These results were used to tailor the mhGAP-IG for the rural Kenyan context.
**Bitta et al. (2020) (143)**	Kenya	Case study	N/A	The authors conducted situational analysis, stakeholder engagement, local adaptation of mhGAP-IG and pilot testing. Barriers to mhGAP-IG implementation included few mental health specialists for referral, unstable medication supplies, difficulties translating the IG into Kiswahili, lack of clarity about organisational roles, unwillingness of health workers to collaborate with THPs to enhance referrals. Stakeholders recommended removing child and adolescent mental health and dementia from the adapted IG.<br>Pilot testing of the adapted IG led to improvements in post-training knowledge scores.
**Hanlon et al. (2016) (144)**	Ethiopia<br>Uganda<br>South Africa<br>India<br>Nepal	Comparative case study	Participants in formative interviews and focus groups contributing to MHCP development:<br>58 (Ethiopia)<br>43 (India)<br>117 (Nepal)<br>133 (South Africa)<br>97 (Uganda)	The authors performed a comparative analysis of district-level mental healthcare plans (MHCPs), including implementation of the mhGAP-IG. Each country selected a sub-group of mhGAP-IG mental, neurological and substance use disorders to prioritise for initial implementation, considering the needs of local districts as well as national priorities.<br>A dedicated mhGAP costing tool was used to inform discussions with key stakeholders about making MHCPs feasible and scalable.<br>Countries differed by the cadre of professionals being trained (e.g. non-mental health specialists in Ethiopia and South Africa). Uganda and South Africa used a Training of Trainers model whilst Ethiopia, India and Nepal used psychiatrist trainers.<br>South Africa adapted its own primary care mental health guidelines to accommodate mhGAP, whereas other countries used the mhGAP-IG alone.
**Peterson et al. (2019) (145)**	Ethiopia<br>Uganda<br>South Africa<br>India<br>Nepal	Qualitative study	Key informants (provincial managers, district managers, facility managers and service providers)	PRIME designed mental health care packages for the 6 countries, focused on integrating mental health into PHC. A key component was adapting the mhGAP-IG and training PHC providers to use it.<br>121 key informant Interviews explored obstacles, synergies and implications of scaling up a model of mental healthcare integrated into PHC, according to the CFIR framework.
**Rahul et al. (2016) (146)**	India	Case study	Key stakeholders (state and district-level planners, health service providers, paramedical and front-line workers)	Situational analysis: document review of knowledge about mental health service delivery in the district.<br>Theory of change workshops: opinions of key stakeholders about clinical and health system outcomes required to develop and implement a successful mental health care plan for 3 disorders.<br>The resulting mental health care package included mhGAP-IG treatment guidelines for depression, psychosis, and alcohol use disorders.
**Acharya et al. (2017) (147)**	Nepal	Qualitative study	N/A	The authors describe ethnographic research to identify suitable Nepali terminology for communicating about mhGAP-IG concepts such as depression and post-traumatic stress disorder (PTSD) with lay persons. 'Heart-mind problems' and 'heart-mind-social problems' were acceptable, non-stigmatising terms to communicate generalised psychological distress. 'Unforgettable tragedy' and 'wounded/scarred heart-mind' were acceptable terms for speaking about PTSD.<br>These concepts were used to tailor the mhGAP-IG for the Nepali context.

Authors	Country	Study design	Sample	Summary of findings
**Doherty et al. (2020) (148)**	Sri Lanka	Qualitative study	5 medical students, 3 lecturers, 2 doctors, 1 director, 1 cameraman	The authors conducted in-depth individual interviews with professionals who worked on adapting the mhGAP-IG training videos for the Sri Lankan context. Most participants emphasised the difficulty of translating mental health concepts into the local Tamil dialect, and role modelling mental health skills in a manner faithful to the source material while still representative of clinical practice in Sri Lanka. Participants struggled with budgeting, logistical difficulties, team cooperation and creative differences, but most experienced the project positively.

Table 8: Summary of included studies using the mhGAP-IG to conduct economic analysis

Authors	Country	Study design	Study details	Summary of findings
**Charlson et al. (2016) (149)**	Syria	Workforce prediction	Prediction of human resource requirements over 15 years based on GBD study prevalence estimates for PTSD and depression and the mhGAP costing tool.	The authors estimated 2.2 million cases of PTSD in Syria and 1.1 million cases of depression, equating to 13.4 and 9.2 YLDs per 1000 population, respectively, requiring 7.6 full time equivalent staff per 100,000 population after scale up, from a baseline of 0.3 per 100,000.
**Charlson et al. (2014) (150)**	48 sub-Saharan African countries	Workforce prediction	Prediction of mental health workforce requirements over 40 years based on epidemiological changes and Global Burden of Disease (GBD) study prevalence estimates using the mhGAP costing tool.	The authors predicted a 130% increase in the burden of mental and substance use disorders in sub-Saharan Africa between 2010 and 2050, causing 45 million years lived with a disability (YLDs), requiring an increase in the mental health workforce of 216,000 full time equivalent staff.
**Hailemichael et al. (2019) (151)**	Ethiopia	Cross-sectional study	A comparative cross-sectional community household survey of 290 households including a person with an mhGAP-IG-diagnosed severe mental disorder and 289 comparison households without a person with severe mental disorder,	Catastrophic health expenditure was defined as annual out of pocket health expenditures exceeding 40% of the household's annual non-food expenditure. Catastrophic health expenditure affected 32% of households with a relative with severe mental disorder and 18% of households without a relative with severe mental disorder. Households including a person with severe mental disorder were more likely to be less satisfied with their financial status, perceive their income as insufficient and to employ hardship coping strategies such as reducing medical visits, food consumption or withdrawing children from school.
**Charlson et al. (2015)(152)**	Pacific Island Countries	Workforce prediction	Projection of mental health workforce requirements over 40 years based on population and burden of disease data and the mhGAP costing tool.	If prevalence rates remain constant, the authors projected a 74% increase in disability-related burden for mental disorders, associated with an anticipated 58% population rise. Projected increases in workforce requirements over the period from 2010-20150 were over 1,300 mental health professionals across all countries.
**Chisholm et al. (2016) (153)**	36 countries across all 6 WHO major regions	Cost-benefit analysis	Global return on investment analysis to compare mhGAP-recommended treatment costs for depression and anxiety disorders between 2016 and 2030 with increases in gross domestic product (GDP) and labour participation resulting from treatment.	The authors estimated that scaling up effective treatment coverage for depression and anxiety disorders between 2016 and 2030 would cost $147 billion. Treatment would create 43 million additional years of healthy life, associated with increased economic productivity of $230 billion for depression and $169 billion for anxiety disorders.
**Chisholm et al. (2017) (154)**	Ethiopia<br>India<br>Nepal<br>Nigeria<br>South Africa<br>Uganda	Cost-benefit analysis	Development of a module for the United Nations' strategic planning tool (OneHealth) to identify the health system resources required to scale up services for psychosis, depression and epilepsy and their associated health gains.	The study calculated that delivering mhGAP-IG recommended care for psychosis, depression and epilepsy in Ethiopia, India, Nepal and Uganda would cost $0.14 – 1.27 per head of population per year. The figure in Nigeria and South Africa was around $5. Such care would yield between 291 and 947 healthy life years per million people.
**Chisholm et al. (2020) (155)**	Ethiopia<br>India<br>Nepal<br>South Africa<br>Uganda	Cohort study	A multi-country intervention cohort study of people with alcohol use disorders, depression, psychosis or epilepsy (in three countries), receiving mhGAP-IG-based treatment. The authors calculated health service, travel and time costs to households at baseline and after 12 months' treatment and measured functional impairment.	The authors found that higher costs of treatment were associated with significantly greater functional impairment in Ethiopia and India, and that higher and out of pocket expenditure was associated with significantly greater functional impairment in India and Uganda.<br>Following initiation of mhGAP-IG-based treatment, service costs and out of pocket expenditure were lower in Ethiopia, South Africa, Uganda but higher in India, Nepal.

Table 9: Summary of included studies reporting use of the mhGAP-IG for other educational purposes

Authors	Country	Study design	Sample	Study details	Summary of findings
**Michael et al. (2020) (156)**	Global	Novel application: competency development	N/A	Description of a new set of mhGAP-IG clinician competencies. The article describes how using outcomes to inform mhGAP training curricula and assessment will focus learning on the knowledge, skills and attitudes required to perform specific tasks.	The authors identified core competencies of an attitude of respect and dignity towards people with mental, neurological and substance use disorders, skills to assess and deliver psychosocial and pharmacological interventions, assess and manage emergency presentations and communicate effectively. Each competency was broken down into specific tasks, standardised and mapped to each mhGAP-IG module.
**Kopchak et al. (2020) (157)**	Ukraine	Novel application: curriculum development	Kyiv Medical University students	The undergraduate psychiatry curriculum was revised using the mhGAP-IG. Didactic teaching hours were reduced, interactive teaching methods were introduced, students were encouraged to self-study using online mhGAP resources and progress was assessed using clinical case discussions.	Benefits of incorporating mhGAP-IG content into pre-service training include sustainability, cost effectiveness, developing a common understanding across health professions and strengthening mental healthcare.
**Maulik et al. (2016) (158)**	India	Novel application: mobile-based electronic decision support systems (EDSS) development	4 Accredited Social Health Activists (ASHAs) and 1 primary care doctors from 3 villages	One EDSS for ASHAs comprised a screening tool based on the PHQ-9 and GAD-7.<br>The other EDSS for primary care doctors used the mhGAP-IG modules for depression, suicide and self-harm, and other emotional/ medically unexplained complaints. Also conducted community anti-stigma campaign.	EDSS were acceptable but required some modifications. Stigma and lack of knowledge about CMDs in the community were noted. Faith and traditional healers were considered important mental health service providers.
**Praharso et al. (2020) (159)**	Indonesia	Cross-sectional study	153 health professionals, comprising 78 mental health professionals and 75 non-mental health practitioners	Staff members completed a self-report questionnaire followed by a semi-structured interview in which they were asked questions about the nature and management of depressive or psychotic symptoms of a patient case vignette. Responses were coded using a list of items based on the mhGAP-IG.	Mental health professionals were significantly less likely to hold stigmatised views towards individuals with mental health problems and significantly more likely to identify symptoms of depression and psychosis and recommend evidence-based treatment.
**Subba et al. (2017) (160)**	Nepal	Novel application: community Informant Detection Tool (CIDT) development.	25 mental health professionals<br>113 community informants<br>(8 interviews + 105 surveys)	Case vignettes and illustrations displaying local idioms of distress for symptoms of depression, psychosis, alcohol use problems, epilepsy and child behavioural problems were created using the mhGAP-IG, with the expert panel of Nepali mental health specialists. A draft tool was evaluated using focus group discussions (n=19) and in-depth interviews (n=6).<br>Applicability of the tool was assessed by practising the CIDT during in-depth interviews with community informants. Community informants completed surveys to assess feasibility of the CIDT.	Community detection of mental, neurological and substance use disorders using the CIDT was considered to be useful and feasible by community stakeholders.

Authors	Country	Study design	Sample	Study details	Summary of findings
**Jordans et al. (2020) (161)**	Nepal	Cluster randomised controlled trial	Female community health volunteers at 24 health facilities	Community volunteers received either standard mental health training or standard training plus CIDT (see above), following random allocation of their health facility.	At health facilities where CIDT was included in training, 309 patients were diagnosed with depression, psychosis, alcohol use disorder or epilepsy. At health facilities where CIDT was not included in training, 182 patients received these diagnoses at registration. The median number of diagnoses was 47% greater at CIDT-trained health centres. Differences remained significant after adjustment for health facilities' population catchment sizes.
**Mills & Hilberg 2019 (162)**	N/A	Qualitative study	N/A	A sociological analysis of the mhGAP-IG's 'living history' and 'social lives' (performance and conditions of possibility). Participant observations were made of different applications of mhGAP, including at the mhGAP Forum, discussions with mhGAP authors and analysis of mhGAP reports.	"Quantification…, decontextualisation, epistemological exclusivity, and digitisation, converge to construct a globalised notion of mental health, out of which a particular algorithmic imaginary of mental disorder emerges."

1. de la Barra F, Irarrazaval M, Valdes A, Soto-Brandt G. Evolving child and adolescent mental health and development programs in Chile. Revista Panamericana De Salud Publica-Pan American Journal of Public Health. 2019;43.
2. Diez-Canseco F, Toyama M, Ipince A, Perez-Leon S, Cavero V, Araya R, et al. Integration of a technology-based mental health screening program into routine practices of primary health care services in Peru (The Allillanchu Project): Development and implementation. Journal of Medical Internet Research. 2018;20(3).
3. Organization WH. Enhancing mental health pre-service training with the mhGAP intervention guide: experiences and lessons learned. 2020.
4. Chaulagain A, Pacione L, Abdulmalik J, Hughes P, Oksana K, Chumak S, et al. WHO Mental Health Gap Action Programme Intervention Guide (mhGAP-IG): The first pre-service training study. International Journal of Mental Health Systems. 2020;14 (1) (no pagination)(47).
5. Villamil-Salcedo V, Vargas-Terrez BE, Díaz-Anzaldúa A. Collaborative Care model in mental health. Scope and experiences after three years of activity in Mexico City. Primary Health Care Research and Development. 2017;18(3):227-34.
6. Robles R, Lopez-Garcia P, Miret M, Cabello M, Cisneros E, Rizo A, et al. WHO-mhGAP training in Mexico: increasing knowledge and readiness for the identification and management of depression and suicide risk in primary care. Archives of Medical Research. 2019;50(8):558-66.
7. Hughes P, Thomson S. mhGAP – the global scenario. Progress in Neurology and Psychiatry. 2019;23(4):4-6.
8. Reginald Fils-Aime J, Grelotti DJ, Thérosme T, Kaiser BN, Raviola G, Alcindor Y, et al. A mobile clinic approach to the delivery of community-based mental health services in rural Haiti. PLoS ONE. 2018;13(6).
9. Hughes P, Hijazi Z, Saeed K. Improving access to mental healthcare for displaced Syrians: case studies from Syria, Iraq and Turkey. BJPsych International. 2016;13(4):84-6.
10. Karaoğlan Kahiloğulları A, Alataş E, Ertuğrul F, Malaj A. Responding to mental health needs of Syrian refugees in Turkey: mhGAP training impact assessment. International Journal of Mental Health Systems. 2020;14(1).
11. Kerbage H, Marranconi F, Chamoun Y, Brunet A, Richa S, Zaman S. Mental Health Services for Syrian Refugees in Lebanon: Perceptions and Experiences of Professionals and Refugees. Qualitative Health Research. 2020;30(6):849-64.
12. van Ommeren M, Hanna F, Weissbecker I, Ventevogel P. Mental health and psychosocial support in humanitarian emergencies. Eastern Mediterranean Health Journal. 2015;21(7):498-502.
13. Sharifi V, Shahrivar Z, Zarafshan H, Ashkezary SB, Stuart E, Mojtabai R, et al. Collaborative care for child and youth mental health problems in a middle-income country: Study protocol for a randomized controlled trial training general practitioners. Trials. 2019;20(1).
14. Spagnolo J, Champagne F, Leduc N, Melki W, Piat M, Laporta M, et al. "We find what we look for, and we look for what we know": factors interacting with a mental health training program to influence its expected outcomes in Tunisia. BMC Public Health. 2018;18(1398).
15. Spagnolo J, Champagne F, Leduc N, Melki W, Bram N, Guesmi I, et al. A program to further integrate mental health into primary care: lessons learned from a pilot trial in Tunisia. 2019;3.
16. Spagnolo J, Champagne F, Leduc N, Rivard M, Melki W, Piat M, et al. Building capacity in mental health care in low- and middle-income countries by training primary care physicians using the mhGAP: a randomized controlled trial. Health Policy and Planning. 2020;35(2):186-98.
17. Kamara S, Walder A, Duncan J, Kabbedijk A, Hughes P, Muana A. Mental health care during the ebola virus disease outbreak in Sierra Leone. Bulletin of the World Health Organization. 2017;95(12):842-7.
18. Echeverri C, Le Roy J, Worku B, Ventevogel PJGMH. Mental health capacity building in refugee primary health care settings in Sub-Saharan Africa: impact, challenges and gaps. 2018;5.
19. Iheanacho T, Marienfeld C, Stefanovics E, Rosenheck RA. Attitudes toward mental illness and changes associated with a brief educational intervention for medical and nursing students in Nigeria. Academic Psychiatry. 2014;38(3):320-4.
20. Ryan GK, Nwefoh E, Aguocha C, Ode PO, Okpoju SO, Ocheche P, et al. Partnership for the implementation of mental health policy in Nigeria: A case study of the Comprehensive Community Mental Health Programme in Benue State. International Journal of Mental Health Systems. 2020;14 (1) (no pagination)(10).
21. Ali S, Saeed K, Hughes P. Evaluation of a mental health training project in the Republic of the Sudan using the Mental Health Gap Action Programme curriculum. International psychiatry : bulletin of the Board of International Affairs of the Royal College of Psychiatrists. 2012;9(2):43-5.
22. Aboaja A, Myles P, Hughes P. Mental health e-supervision for primary care doctors in Sudan using the WHO mhGAP Intervention Guide. BJPsych International. 2015;12(S1):S16-S9.

23. Osman AHM, Bakhiet A, Elmusharaf S, Omer A, Abdelrahman A. Sudan's mental health service: Challenges and future horizons. BJPsych International. 2020;17(1):17-9.
24. Gavaghan L, Hughes P, Saeed K, Whitwell S. Teaching psychiatry to undergraduate medical students in Somalia. International psychiatry : bulletin of the Board of International Affairs of the Royal College of Psychiatrists. 2014;11(1):15-7.
25. Murphy R, Clissold E, Keynejad RC. Problem-based, peer-to-peer global mental health e-learning between the UK and Somaliland: a pilot study. Evidence-based mental health. 2017;20(4):142-6.
26. Ayano G, Assefa D, Haile K, Chaka A, Haile K, Solomon M, et al. Mental health training for primary health care workers and implication for success of integration of mental health into primary care: evaluation of effect on knowledge, attitude and practices (KAP). International Journal of Mental Health Systems. 2017;11.
27. Catalao R, Eshetu T, Tsigebrhan R, Medhin G, Fekadu A, Hanlon C. Implementing integrated services for people with epilepsy in primary care in Ethiopia: a qualitative study. BMC health services research. 2018;18(1):372.
28. Selamu M, Hanlon C, Medhin G, Thornicroft G, Fekadu AJHrfh. Burnout among primary healthcare workers during implementation of integrated mental healthcare in rural Ethiopia: a cohort study. 2019;17(1):58.
29. Mutiso VN, Musyimi CW, Rebello TJ, Gitonga I, Tele A, Pike KM, et al. Patterns of concordances in mhGAP-IG screening and DSM-IV/ICD10 diagnoses by trained community service providers in Kenya: A pilot cross-sectional study. [References]: Social Psychiatry and Psychiatric Epidemiology: The International Journal for Research in Social and Genetic Epidemiology and Mental Health Services. Vol.53(11), 2018, pp. 1277-1287.; 2018.
30. Mutiso VN, Pike KM, Musyimi CN, Rebello TJ, Telel A, Gitonga I, et al. Changing patterns of mental health knowledge in rural Kenya after intervention using the WHO mhGAP-Intervention Guide. Psychological Medicine. 2019;49(13):2227-36.
31. Mutiso VN, Musyimi CW, Rebello TJ, Ogutu MO, Ruhara R, Nyamai D, et al. Perceived impacts as narrated by service users and providers on practice, policy and mental health system following the implementation of the mhGAP-IG using the TEAM model in a rural setting in Makueni County, Kenya: A qualitative approach. International Journal of Mental Health Systems. 2019;13 (1) (no pagination)(56).
32. Mutiso VN, Pike K, Musyimi CW, Rebello TJ, Tele A, Gitonga I, et al. Feasibility of WHO mhGAP-intervention guide in reducing experienced discrimination in people with mental disorders: A pilot study in a rural Kenyan setting. [References]: Epidemiology and Psychiatric Sciences. Vol.28(2), 2019, pp. 156-167.; 2019.
33. Akol A, Babirye JN, Makumbi F, Engebretsen IMS. Effect of primary health care worker training on identification of child-and adolescent mental health conditions: A randomised controlled trial in Uganda. Tropical Medicine and International Health. 2017;22 (Supplement 1):283.
34. Akol A, Makumbi F, Babirye JN, Nalugya JS, Nshemereirwe S, Engebretsen IMS. Does mhGAP training of primary health care providers improve the identification of child- and adolescent mental, neurological or substance use disorders? Results from a randomized controlled trial in Uganda. Global Mental Health. 2018;5.
35. Akol A, Nalugya J, Nshemereirwe S, Babirye JN, Engebretsen IMS. Does child and adolescent mental health in-service training result in equivalent knowledge gain among cadres of non-specialist health workers in Uganda? A pre-test post-test study. International Journal of Mental Health Systems. 2017;11.
36. Ahrens J, Kokota D, Mafuta C, Konyani M, Chasweka D, Mwale O, et al. Implementing an mhGAP-based training and supervision package to improve healthcare workers' competencies and access to mental health care in Malawi. International Journal of Mental Health Systems. 2020;14 (1) (no pagination)(11).
37. Kokota D, Lund C, Ahrens J, Breuer E, Gilfillan S. Evaluation of mhGAP training for primary healthcare workers in Mulanje, Malawi: A quasi-experimental and time series study. International Journal of Mental Health Systems. 2020;14 (1) (no pagination)(3).
38. Patel AA, Wibecan L, Tembo O, Kalyelye P, Mathews M, Ciccone O. Improving paediatric epilepsy management at the first level of care: A pilot education intervention for clinical officers in Zambia. BMJ Open. 2019;9(7).
39. Patel AA, Ciccone O, Nkole KL, Kalyelye P, Sham L, Kielian A, et al. Development and Evaluation of a Pediatric Epilepsy Training Program for First Level Providers in Zambia. Global Pediatric Health. 2020;7.
40. Sibeko G, Milligan PD, Roelofse M, Molefe L, Jonker D, Ipser J, et al. Piloting a mental health training programme for community health workers in South Africa: An exploration of changes in knowledge, confidence and attitudes. [References]: BMC Psychiatry. Vol.18 2018, ArtID 191.; 2018.
41. Gumber R, Gangavati S, Bhaumik S, Cooray S, Purandare K, Mendis J, et al. The WHO mhGAP Intervention Guide for people with intellectual disability: The Sri Lankan experience. BJPsych International. 2015;12(S1):S19-S23.
42. Doherty S. Integrating mental health into primary care for conflict-affected internal forced migrants in Northern Sri Lanka (COMGAP-S). ISRCTN Registry. 2017.

43. Humayun A, Azad N, ul Haq I, Khan FR, Ahmad A, Farooq RK. Mental health and psychosocial support for the internally displaced persons in Bannu, Pakistan. Intervention-International Journal of Mental Health Psychosocial Work and Counselling in Areas of Armed Conflict. 2016;14(1):33-49.
44. Pathare S, Shields-Zeeman L, Vijayakumar L, Pandit D, Nardodkar R, Chatterjee S, et al. Evaluation of the SPIRIT Integrated Suicide Prevention Programme: study protocol for a cluster-randomised controlled trial in rural Gujarat, India. Trials. 2020;21(1):572.
45. Dyer G, Biswas M. Psychological and psychiatric care for Rohingya refugees in Bangladesh. Intervention. 2019;17(2):217-24.
46. Momotaz H, Ahmed HU, Uddin MMJ, Karim R, Khan MA, Al-Amin R, et al. Implementing the Mental Health Gap Action Programme in Cox's Bazar, Bangladesh. Intervention-International Journal of Mental Health Psychosocial Work and Counselling in Areas of Armed Conflict. 2019;17(2):243-51.
47. Chase LE, Marahatta K, Sidgel K, Shrestha S, Gautam K, Luitel NP, et al. Building back better? Taking stock of the post-earthquake mental health and psychosocial response in Nepal. [References]: International Journal of Mental Health Systems. Vol.12 2018, ArtID 44.; 2018.
48. Rai S, Gurung D, Kaiser BN, Sikkema KJ, Dhakal M, Bhardwaj A, et al. A service user co-facilitated intervention to reduce mental illness stigma among primary healthcare workers: Utilizing perspectives of family members and caregivers. [References]: Families, Systems, & Health. Vol.36(2), 2018, pp. 198-209.; 2018.
49. Kohrt BA, Jordans MJD, Turner EL, Sikkema KJ, Luitel NP, Rai S, et al. Reducing stigma among healthcare providers to improve mental health services (RESHAPE): Protocol for a pilot cluster randomized controlled trial of a stigma reduction intervention for training primary healthcare workers in Nepal. Pilot and Feasibility Studies. 2018;4 (1) (no pagination)(36).
50. Kohrt BA, Mutamba BB, Luitel NP, Gwaikolo W, Onyango Mangen P, Nakku J, et al. How competent are non-specialists trained to integrate mental health services in primary care? Global health perspectives from Uganda, Liberia, and Nepal. [References]: International Review of Psychiatry. Vol.30(6), 2018, pp. 182-198.; 2018.
51. Rajbhandari H, Joshi S, Malakar S, Paudel P, Jain P, Uppadaya K, et al. Epilepsy field workers, a smartphone application and telephone telemedicine: Safe and effective epilepsy care in rural Nepal. Seizure. 2019;64:54-8.
52. Kohrt BA, Turner EL, Rai S, Bhardwaj A, Sikkema KJ, Adelekun A, et al. Reducing mental illness stigma in healthcare settings: Proof of concept for a social contact intervention to address what matters most for primary care providers. Social Science and Medicine. 2020;250 (no pagination)(112852).
53. Li J, Li J, Thornicroft G, Yang H, Chen W, Huang Y. Training community mental health staffin Guangzhou, China: Evaluation of the effect of a new training model. BMC Psychiatry. 2015;15(1).
54. Li J, Fan Y, Zhong HQ, Duan XL, Chen W, Evans-Lacko S, et al. Effectiveness of an anti-stigma training on improving attitudes and decreasing discrimination towards people with mental disorders among care assistant workers in Guangzhou, China. International Journal of Mental Health Systems. 2019;13(1).
55. de Moraes Weintraub ACA, Garcia MG, Birri E, Severy N, Ferir MC, Ali E, et al. Not forgetting severe mental disorders in humanitarian emergencies: A descriptive study from the Philippines. International Health. 2016;8(5):336-44.
56. Ryan G, Iemmi V, Hanna F, Loryman H, Eaton J. Mental health for sustainable development: a topic guide for development professionals. 2020.
57. Charlson F, Chang O, Kubuabola I, Schess J, Latu C, Hunter E, et al. Implementation of the mental health Gap Action Programme (mhGAP) within the Fijian Healthcare System: A mixed-methods evaluation. International Journal of Mental Health Systems. 2019;13 (1) (no pagination)(43).
58. Setoya Y, Kestel D. WHO Mental Health Gap Action Programme implementation in the Small Island Development States: Experience from the Pacific and English-speaking Caribbean countries. BJPsych International. 2018;15(2):27-30.
59. Usher K, Park T, Trueman S, Redman-Maclaren M, Casella E, Woods C. An educational program for mental health nurses and community health workers from pacific island countries: Results from a pilot study. Issues in Mental Health Nursing. 2014;35(5):337-43.
60. Miguel-Esponda G, Bohm-Levine N, Rodriguez-Cuevas FG, Cohen A, Kakuma R. Implementation process and outcomes of a mental health programme integrated in primary care clinics in rural Mexico: A mixed-methods study. International Journal of Mental Health Systems. 2020;14 (1) (no pagination)(21).
61. Miguel Esponda G. What enables and hinders implementation? A mixed-methods case study of a mental health programme implemented in primary care clinics in rural Mexico: London School of Hygiene & Tropical Medicine; 2020.

62. Gureje O, Oladeji BD, Araya R, Montgomery AA, Kola L, Kirmayer L, et al. Expanding care for perinatal women with depression (EXPONATE): Study protocol for a randomized controlled trial of an intervention package for perinatal depression in primary care. BMC Psychiatry. 2015;15(1).
63. Gureje O, Oladeji BD, Araya R, Montgomery AA. A cluster randomized clinical trial of a stepped care intervention for depression in primary care (STEPCARE)- study protocol. BMC Psychiatry. 2015;15(1).
64. Gureje O, Makanjuola V, Kola L, Yusuf B, Price L, Esan O, et al. COllaborative Shared care to IMprove Psychosis Outcome (COSIMPO): Study protocol for a randomized controlled trial. Trials. 2017;18(1).
65. Gureje O, Seedat S, Kola L, Appiah-Poku J, Othieno C, Harris B, et al. Partnership for mental health development in Sub-Saharan Africa (PaM-D): A collaborative initiative for research and capacity building. Epidemiology and Psychiatric Sciences. 2019;28(4):389-96.
66. Gureje O, Oladeji BD, Montgomery AA, Araya R, Bello T, Chisholm D, et al. High- versus low-intensity interventions for perinatal depression delivered by non-specialist primary maternal care providers in Nigeria: Cluster randomised controlled trial (the EXPONATE trial). [References]: The British Journal of Psychiatry. Vol.215(3), 2019, pp. 528-535.; 2019.
67. Gureje O, Oladeji BD, Montgomery AA, Bello T, Kola L, Ojagbemi A, et al. Effect of a stepped-care intervention delivered by lay health workers on major depressive disorder among primary care patients in Nigeria (STEPCARE): a cluster-randomised controlled trial. Lancet Global Health. 2019;7(7):e951-e60.
68. Adewuya AO, Momodu O, Olibamoyo O, Adegbaju A, Adesoji O, Adegbokun A. The effectiveness and acceptability of mobile telephone adherence support for management of depression in the Mental Health in Primary Care (MeHPriC)project, Lagos, Nigeria: A pilot cluster randomised controlled trial. Journal of Affective Disorders. 2019;253:118-25.
69. Adewuya AO, Adewumi T, Momodu O, Olibamoyo O, Adesoji O, Adegbokun A, et al. Development and feasibility assessment of a collaborative stepped care intervention for management of depression in the mental health in primary care (MeHPriC) project, Lagos, Nigeria. Psychological Medicine. 2019;49(13):2149-57.
70. Adewuya AO, Ola BA, Coker O, Atilola O, Fasawe A, Ajomale T. A stepped care intervention for non-specialist health workers' management of depression in the Mental Health in Primary Care (MeHPriC) project, Lagos, Nigeria: A cluster randomised controlled trial. General Hospital Psychiatry. 2019;60:76-82.
71. Gureje O, Kola L, Oladeji BD, Abdulmalik J, Ayinde O, Zelkowitz P, et al. Responding to the challenge of Adolescent Perinatal Depression (RAPiD): Protocol for a cluster randomized hybrid trial of psychosocial intervention in primary maternal care. Trials. 2020;21 (1) (no pagination)(231).
72. Hailemariam M, Fekadu A, Medhin G, Prince M, Hanlon C. Equitable access to mental healthcare integrated in primary care for people with severe mental disorders in rural Ethiopia: A community-based cross-sectional study. International Journal of Mental Health Systems. 2019;13(1).
73. Habtamu K, Medhin G, Selamu M, Tirfessa K, Hanlon C, Fekadu A. Functional impairment among people diagnosed with depression in primary healthcare in rural Ethiopia: A comparative cross-sectional study. International Journal of Mental Health Systems. 2019;13 (1) (no pagination)(50).
74. Forthal S, Fekadu A, Medhin G, Selamu M, Thornicroft G, Hanlon C. Rural vs urban residence and experience of discrimination among people with severe mental illnesses in Ethiopia. BMC Psychiatry. 2019;19(1).
75. Hanlon C, Medhin G, Selamu M, Birhane R, Dewey M, Tirfessa K, et al. Impact of integrated district level mental health care on clinical and social outcomes of people with severe mental illness in rural Ethiopia: An intervention cohort study. Epidemiology and Psychiatric Sciences. 2019.
76. Tirfessa K, Lund C, Medhin G, Selamu M, Birhane R, Hailemichael Y, et al. Impact of integrated mental health care on food insecurity of households of people with severe mental illness in a rural African district: a community-based, controlled before–after study. Tropical Medicine and International Health. 2020;25(4):414-23.
77. Musyimi CW, Mutiso VN, Ndetei DM, Unanue I, Desai D, Patel SG, et al. Mental health treatment in Kenya: Task-sharing challenges and opportunities among informal health providers. International Journal of Mental Health Systems. 2017;11(1).
78. Musyimi C. Engaging Traditional Birth Attendants to Reduce Maternal Depression in Rural Kenya. ClinicalTrialsgov. 2017.
79. Mutiso V, Musyimi C, Tele A, Gitonga I, Ndetei D. Feasibility study on the mhGAP-IG as a tool to enhance parental awareness of symptoms of mental disorders in lower primary (6-10 year old) school-going children: Towards inclusive child mental health services in a Kenyan setting. Early intervention in psychiatry. 2020;14:14.
80. Mutiso VN, Pike KM, Musyimi CW, Gitonga I, Tele A, Rebello TJ, et al. Feasibility and effectiveness of nurses and clinical officers in implementing the WHO mhGAP intervention guide: Pilot study in Makueni County, Kenya. [References]: General Hospital Psychiatry. Vol.59 2019, pp. 20-29.; 2019.

81. Kumar M, Huang KY, Othieno C, Wamalwa D, Hoagwood K, Unutzer J, et al. Implementing combined WHO mhGAP and adapted group interpersonal psychotherapy to address depression and mental health needs of pregnant adolescents in Kenyan primary health care settings (INSPIRE): a study protocol for pilot feasibility trial of the integrated intervention in LMIC settings. Pilot and Feasibility Studies. 2020;6 (1) (no pagination)(136).
82. Mpango R. Integrating depression management in HIV care in Uganda. ISRCTN Registry. 2017.
83. Nakku JEM, Rathod SD, Garman EC, Ssebunnya J, Kangere S, De Silva M, et al. Evaluation of the impacts of a district-level mental health care plan on contact coverage, detection and individual outcomes in rural Uganda: A mixed methods approach. International Journal of Mental Health Systems. 2019;13(1).
84. Smith SL, Misago CN, Osrow RA, Franke MF, Iyamuremye JD, Dusabeyezu JD, et al. Evaluating process and clinical outcomes of a primary care mental health integration project in rural Rwanda: A prospective mixed-methods protocol. BMJ Open. 2017;7(2).
85. Smith SL, Franke MF, Rusangwa C, Mukasakindi H, Nyirandagijimana B, Bienvenu R, et al. Outcomes of a primary care mental health implementation program in rural Rwanda: A quasi-experimental implementation-effectiveness study. PLoS ONE. 2020;15(2).
86. Dos Santos PF, Cumbe V, Gouveia ML, De Fouchier C, Teuwen D, Dua T. Implementation of mhGAP in Mozambique: Integrating epilepsy care into the primary health care system. International Journal of Mental Health Systems. 2019;13 (1) (no pagination)(36).
87. Madhombiro M, Dube B, Dube M, Zunza M, Chibanda D, Rusakaniko S, et al. Intervention for alcohol use disorders at an HIV care clinic in Harare: a pilot and feasibility study. Addiction science & clinical practice. 2019;14(1):16.
88. Fairall L, Petersen I, Zani B, Folb N, Georgeu-Pepper D, Selohilwe O, et al. Collaborative care for the detection and management of depression among adults receiving antiretroviral therapy in South Africa: Study protocol for the CobALT randomised controlled trial. Trials. 2018;19(1).
89. Baron EC, Rathod SD, Hanlon C, Prince M, Fedaku A, Kigozi F, et al. Impact of district mental health care plans on symptom severity and functioning of patients with priority mental health conditions: The Programme for Improving Mental Health Care (PRIME) cohort protocol. BMC Psychiatry. 2018;18(1).
90. Hamdani SU, Akhtar P, Zill EH, Nazir H, Minhas FA, Sikander S, et al. WHO Parent Skills Training (PST) programme for children with developmental disorders and delays delivered by Family Volunteers in rural Pakistan: Study protocol for effectiveness implementation hybrid cluster randomized controlled trial. Global Mental Health. 2017;4(e11).
91. Robinson M, Haq Z, Farooq S. Supervised treatment for outpatients with schizophrenia (STOPS+). ISRCTN Registry. 2019.
92. Maulik PK, Kallakuri S, Devarapalli S, Vadlamani VK, Jha V, Patel A. Increasing use of mental health services in remote areas using mobile technology: A pre- post evaluation of the SMART Mental Health project in rural India. Journal of Global Health. 2017;7(1).
93. Elshazly M, Budosan B, Mahmudul Alam ANM, Khan N, Ventevogel P. Challenges and opportunities for Rohingya mental health and psychosocial support programming. Intervention. 2019;17(2):197-205.
94. Tarannum S, Elshazly M, Harlass S, Ventevogel P. Integrating mental health into primary health care in Rohingya refugee settings in Bangladesh: experiences of UNHCR. Intervention-International Journal of Mental Health Psychosocial Work and Counselling in Areas of Armed Conflict. 2019;17(2):130-9.
95. Jha A, Sapkota N. Dementia assessment and management protocol for doctors in Nepal. Journal of the Nepal Medical Association. 2013;52(5):292-8.
96. Jordans MJD, Kohrt BA, Luitel NP, Lund C, Komproe IH. Proactive community case-finding to facilitate treatment seeking for mental disorders, Nepal. Bulletin of the World Health Organization. 2017;95(7):531-6.
97. Breuer E, Subba P, Luitel N, Jordans M, Silva Md, Marchal B, et al. Using qualitative comparative analysis and Theory of Change to unravel the effects of a mental health intervention on service utilisation in Nepal. BMJ Global Health. 2018;3(6).
98. Jordans MJD, Luitel NP, Kohrt BA, Rathod SD, Garman EC, De Silva M, et al. Community-, facility-, and individual-level outcomes of a district mental healthcare plan in a low-resource setting in Nepal: A population-based evaluation. PLoS Medicine. 2019;16(2).
99. Jordans MJD, Luitel NP, Garman E, Kohrt BA, Rathod SD, Shrestha P, et al. Effectiveness of psychological treatments for depression and alcohol use disorder delivered by community-based counsellors: Two pragmatic randomised controlled trials within primary healthcare in Nepal. British Journal of Psychiatry. 2019;215(2):485-93.
100. Jordans MJD, Garman EC, Luitel NP, Kohrt BA, Lund C, Patel V, et al. Impact of integrated district level mental health care on clinical and functioning outcomes of people with depression and alcohol use disorder in Nepal: A non-randomised controlled study. BMC Psychiatry. 2020;20(1).

101. Upadhaya N, Jordans MJD, Adhikari RP, Gurung D, Petrus R, Petersen I, et al. Evaluating the integration of chronic care elements in primary health care for people with mental illness: A longitudinal study in Nepal conducted among primary health care workers. BMC Health Services Research. 2020;20(1).
102. Luitel NP. Treatment coverage, barriers to care and factors associated with help-seeking behaviour of adults with depression and alcohol use disorder in Chitwan district, Nepal: Faculty of Health Sciences; 2020.
103. Luitel NP, Breuer E, Adhikari A, Kohrt BA, Lund C, Komproe IH, et al. Process evaluation of a district mental healthcare plan in Nepal: A mixed-methods case study. BJPsych Open. 2020;6 (4) (no pagination)(e77).
104. Aldridge LR, Garman EC, Luitel NP, Jordans MJD. Impact of a district mental health care plan on suicidality among patients with depression and alcohol use disorder in Nepal. PLoS ONE. 2020;15(4).
105. Kohrt B. E-MhGAP Intervention Guide in Low- and Middle-income Countries: proof-of-concept for Impact and Acceptability. ClinicalTrialsgov. 2020.
106. Anjara SG, Bonetto C, Ganguli P, Setiyawati D, Mahendradhata Y, Yoga BH, et al. Can General Practitioners manage mental disorders in primary care? A partially randomised, pragmatic, cluster trial. PLoS ONE. 2019;14 (11) (no pagination)(e0224724).
107. Powell T, Brack J. Humanitarian response after a complex environmental disaster: A case study of Typhoon Haiyan. Social Work and Sustainability in Asia: Facing the Challenges of Global Environmental Changes2019. p. 109-18.
108. Budosan B, Mahoney J, Dorego WC, Aziz S, Ratnasabapathipillai K. Three models of scaling up mental healthcare post-disaster: common challenges. Intervention. 2020;18(1):18-27.
109. Aiyenigba AO, Weeks AD, Rahman A. Managing psychological trauma of infertility. African Journal of Reproductive Health. 2019;23(2):76-91.
110. Geibel S, Habtamu K, Mekonnen G, Jani N, Kay L, Shibru J, et al. Reliability and validity of an interviewer-administered adaptation of the youth self-report for mental health screening of vulnerable young people in Ethiopia. PLoS ONE. 2016;11(2).
111. Ambaw F, Mayston R, Hanlon C, Alem A. Depression among patients with tuberculosis: Determinants, course and impact on pathways to care and treatment outcomes in a primary care setting in southern Ethiopia - A study protocol. BMJ Open. 2015;5(7).
112. Asher L, De Silva M, Hanlon C, Weiss HA, Birhane R, Ejigu DA, et al. Community-based Rehabilitation Intervention for people with Schizophrenia in Ethiopia (RISE): Study protocol for a cluster randomised controlled trial. Trials. 2016;17(1).
113. Keynejad RC, Bitew T, Bitew T, Sorsdahl K, Myers B, Myers B, et al. Problem solving therapy (PST) tailored for intimate partner violence (IPV) versus standard PST and enhanced usual care for pregnant women experiencing IPV in rural Ethiopia: Protocol for a randomised controlled feasibility trial. Trials. 2020;21(1).
114. Smith Fawzi MC, Siril H, Larson E, Aloyce Z, Araya R, Kaale A, et al. Healthy Options: Study protocol and baseline characteristics for a cluster randomized controlled trial of group psychotherapy for perinatal women living with HIV and depression in Tanzania. BMC Public Health. 2020;20(1).
115. Wainberg ML. Training Providers to Conduct PRIDES-sSA. ClinicalTrialsgov. 2018.
116. Odenwald M, Lingenfelder B, Peschel W, Haibe FA, Warsame AM, Omer A, et al. A pilot study on community-based outpatient treatment for patients with chronic psychotic disorders in Somalia: Change in symptoms, functioning and co-morbid khat use. International Journal of Mental Health Systems. 2012;6.
117. Khan MN, Dherani M, Chiumento A, Atif N, Bristow K, Sikander S, et al. Evaluating feasibility and acceptability of a local psycho-educational intervention for pregnant women with common mental problems affected by armed conflict in Swat, Pakistan: A parallel randomized controlled feasibility trial. International Journal of Social Psychiatry. 2017;63(8):724-35.
118. Atif N, Nazir H, Zafar S, Chaudhri R, Atiq M, Mullany LC, et al. Development of a Psychological Intervention to Address Anxiety During Pregnancy in a Low-Income Country. Frontiers in Psychiatry. 2020;10.
119. Sikander S, Ahmad I, Atif N, Zaidi A, Vanobberghen F, Weiss HA, et al. Delivering the Thinking Healthy Programme for perinatal depression through volunteer peers: a cluster randomised controlled trial in Pakistan. The Lancet Psychiatry. 2019;6(2):128-39.
120. Maselko J, Sikander S, Turner EL, Bates LM, Ahmad I, Atif N, et al. Effectiveness of a peer-delivered, psychosocial intervention on maternal depression and child development at 3 years postnatal: a cluster randomised trial in Pakistan. The Lancet Psychiatry. 2020;7(9):775-87.
121. Surkan PJ, Hamdani SU, Huma ZE, Nazir H, Atif N, Rowther AA, et al. Cognitive-behavioral therapy-based intervention to treat symptoms of anxiety in pregnancy in a prenatal clinic using non-specialist providers in Pakistan: Design of a randomised trial. BMJ Open. 2020;10(4).

122. Nadkarni A, Velleman R, Dabholkar H, Shinde S, Bhat B, McCambridge J, et al. The systematic development and pilot randomized evaluation of counselling for alcohol problems, a lay counselor-delivered psychological treatment for harmful drinking in primary care in India: The PREMIUM study. Alcoholism: Clinical and Experimental Research. 2015;39(3):522-31.
123. Nadkarni A, Weobong B, Weiss HA, McCambridge J, Bhat B, Katti B, et al. Counselling for Alcohol Problems (CAP), a lay counsellor-delivered brief psychological treatment for harmful drinking in men, in primary care in India: a randomised controlled trial. The Lancet. 2017;389(10065):186-95.
124. Nadkarni A, Weiss HA, Weobong B, McDaid D, Singla DR, Park AL, et al. Sustained effectiveness and cost-effectiveness of Counselling for Alcohol Problems, a brief psychological treatment for harmful drinking in men, delivered by lay counsellors in primary care: 12-month follow-up of a randomised controlled trial. PLoS Medicine. 2017;14(9).
125. Patel V, Weobong B, Weiss HA, Anand A, Bhat B, Katti B, et al. The Healthy Activity Program (HAP), a lay counsellor-delivered brief psychological treatment for severe depression, in primary care in India: a randomised controlled trial. The Lancet. 2017;389(10065):176-85.
126. Weobong B, Weiss HA, McDaid D, Singla DR, Hollon SD, Nadkarni A, et al. Sustained effectiveness and cost-effectiveness of the Healthy Activity Programme, a brief psychological treatment for depression delivered by lay counsellors in primary care: 12-month follow-up of a randomised controlled trial. PLoS Medicine. 2017;14(9).
127. Prabhakaran D, Jha D, Prieto-Merino D, Roy A, Singh K, Ajay VS, et al. Effectiveness of an mHealth-Based Electronic Decision Support System for Integrated Management of Chronic Conditions in Primary Care: The mWellcare Cluster-Randomized Controlled Trial. Circulation. 2019;139(3):380-91.
128. Spector A, Stoner CR, Chandra M, Vaitheswaran S, Du B, Comas-Herrera A, et al. Mixed methods implementation research of cognitive stimulation therapy (CST) for dementia in low and middle-income countries: Study protocol for Brazil, India and Tanzania (CST-International). BMJ Open. 2019;9(8).
129. Kohrt B. Reducing Stigma Among Healthcare Providers (RESHAPE-cRCT). ClinicalTrialsgov. 2020.
130. Sangraula M, Turner EL, Luitel NP, Van'T Hof E, Shrestha P, Ghimire R, et al. Feasibility of Group Problem Management plus (PM+) to improve mental health and functioning of adults in earthquake-affected communities in Nepal. Epidemiology and Psychiatric Sciences. 2020.
131. Sangraula M, Van't Hof E, Luitel NP, Turner EL, Marahatta K, Nakao JH, et al. Protocol for a feasibility study of groupbased focused psychosocial support to improve the psychosocial well-being and functioning of adults affected by humanitarian crises in Nepal: Group Problem Management plus (PM+). Pilot and Feasibility Studies. 2018;4(1).
132. Van't Hof E, Sangraula M, Luitel NP, Turner EL, Marahatta K, van Ommeren M, et al. Effectiveness of Group Problem Management Plus (Group-PM plus) for adults affected by humanitarian crises in Nepal: study protocol for a cluster randomized controlled trial. Trials. 2020;21(1).
133. Li J, Huang YG, Ran MS, Fan Y, Chen W, Evans-Lacko S, et al. Community-based comprehensive intervention for people with schizophrenia in Guangzhou, China: Effects on clinical symptoms, social functioning, internalized stigma and discrimination. Asian Journal of Psychiatry. 2018;34:21-30.
134. Tay AK, Mung HK, Miah MAA, Balasundaram S, Ventevogel P, Badrudduza M, et al. An Integrative Adapt Therapy for common mental health symptoms and adaptive stress amongst Rohingya, Chin, and Kachin refugees living in Malaysia: A randomized controlled trial. PLoS Medicine. 2020;17(3).
135. Alhuwail D, Albaj R, Ahmad F, Aldakheel K. The state of mental digi-therapeutics: A systematic assessment of depression and anxiety apps available for Arabic speakers. International Journal of Medical Informatics. 2020;135.
136. Gómez-Carrillo A, Lencucha R, Faregh N, Veissière S, Kirmayer LJ. Engaging culture and context in mhGAP implementation: Fostering reflexive deliberation in practice. BMJ Global Health. 2020;5(9).
137. Faregh N, Lencucha R, Ventevogel P, Dubale BW, Kirmayer LJ. Considering culture, context and community in mhGAP implementation and training: Challenges and recommendations from the field. International Journal of Mental Health Systems. 2019;13 (1) (no pagination)(58).
138. Eaton J, Gureje O, De Silva M, Sheikh TL, Ekpe EE, Abdulaziz M, et al. A structured approach to integrating mental health services into primary care: Development of the Mental Health Scale Up Nigeria intervention (mhSUN). International Journal of Mental Health Systems. 2018;12(1).
139. Spagnolo J, Champagne F, Leduc N, Melki W, Guesmi I, Bram N, et al. Tailoring a training based on the Mental Health Gap Action Programme (mhGAP) Intervention Guide (IG) to Tunisia: process and relevant adaptations. Global Mental Health. 2018;5.
140. Spagnolo J, Gautier L, Champagne F, Leduc N, Melki W, N'Guessan K, et al. Reflecting on knowledge translation strategies from global health research projects in Tunisia and the Republic of Côte d'Ivoire. International Journal of Public Health. 2020.

141. Fekadu A, Hanlon C, Medhin G, Alem A, Selamu M, Giorgis TW, et al. Development of a scalable mental healthcare plan for a rural district in Ethiopia. British Journal of Psychiatry. 2016;208:s4-s12.
142. Bitta MA, Kariuki SM, Gona J, Abubakar A, Newton CRJC. Priority mental, neurological and substance use disorders in rural Kenya: Traditional health practitioners' and primary health care workers' perspectives. [References]: PLoS ONE. Vol.14(3), 2019, ArtID e0220034.; 2019.
143. Bitta MA, Kariuki SM, Omar A, Nasoro L, Njeri M, Kiambu C, et al. Contextualizing and pilot testing the Mental Health Gap Action Programme Intervention Guide (mhGAP-IG) to primary healthcare workers in Kilifi, Kenya. Global Mental Health. 2020;7 (no pagination)(e11).
144. Hanlon C, Fekadu A, Jordans M, Kigozi F, Petersen I, Shidhaye R, et al. District mental healthcare plans for five lowand middle-income countries: Commonalities, variations and evidence gaps. British Journal of Psychiatry. 2016;208:s47-s54.
145. Petersen I, van Rensburg A, Kigozi F, Semrau M, Hanlon C, Abdulmalik J, et al. Scaling up integrated primary mental health in six low-and middle-income countries: obstacles, synergies and implications for systems reform. 2019;5(5).
146. Rahul S, Sanjay S, Vaibhav M, Sandesh S, Shalini A, Rohit R, et al. Development and piloting of a plan for integrating mental health in primary care in Sehore district, Madhya Pradesh, India. (Special Issue: Mental health plans in five low- and middle-income countries: PRogramme for Improving Mental health carE (PRIME).). British Journal of Psychiatry. 2016;208(s56):s13-s20.
147. Acharya B, Basnet M, Rimal P, Citrin D, Hirachan S, Swar S, et al. Translating mental health diagnostic and symptom terminology to train health workers and engage patients in cross-cultural, non-English speaking populations. International Journal of Mental Health Systems. 2017;11(1).
148. Doherty S, Dass G, Edward A, Manolova G, Solomon M. Challenges and lessons learned in re-filming the who mhgap training videos for Sri Lankan context - a qualitative study. Conflict and Health. 2020;14(7).
149. Charlson FJ, Lee YY, Diminic S, Whiteford H. Applications of the epidemiological modelling outputs for targeted mental health planning in conflict-affected populations: the Syria case-study. Global Mental Health. 2016;3.
150. Charlson FJ, Diminic S, Lund C, Degenhardt L, Whiteford HA. Mental and substance use disorders in Sub-Saharan Africa: Predictions of epidemiological changes and mental health workforce requirements for the next 40 years. PLoS ONE. 2014;9(10).
151. Hailemichael Y, Hailemariam D, Tirfessa K, Docrat S, Alem A, Medhin G, et al. Catastrophic out-of-pocket payments for households of people with severe mental disorder: A comparative study in rural Ethiopia. International Journal of Mental Health Systems. 2019;13(1).
152. Charlson FJ, Diminic S, Whiteford HA. The Rising Tide of Mental Disorders in the Pacific Region: Forecasts of Disease Burden and Service Requirements from 2010 to 2050. Asia and the Pacific Policy Studies. 2015;2(2):280-92.
153. Chisholm D, Burman-Roy S, Fekadu A, Kathree T, Kizza D, Luitel NP, et al. Estimating the cost of implementing district mental healthcare plans in five low-and middle-income countries: The PRIME study. British Journal of Psychiatry. 2016;208:s71-s8.
154. Chisholm D, Heslin M, Docrat S, Nanda S, Shidhaye R, Upadhaya N, et al. Scaling-up services for psychosis, depression and epilepsy in sub-Saharan Africa and South Asia: Development and application of a mental health systems planning tool (OneHealth). Epidemiology and Psychiatric Sciences. 2017;26(3):234-44.
155. Chisholm D, Garman E, Breuer E, Fekadu A, Hanlon C, Jordans M, et al. Health service costs and their association with functional impairment among adults receiving integrated mental health care in five low- And middle-income countries- And PRIME cohort study. Health Policy and Planning. 2020;35(5):567-76.
156. Michael S, Chowdhary N, Rawstorne P, Dua T. Developing competencies for the WHO mhGAP Intervention Guide Version 2.0 training package. World Psychiatry. 2020;19(2):248-9.
157. Kopchak O, Pinchuk I, Ivnev B, Skokauskas N. Reforming Undergraduate Psychiatry Training in Ukraine. Journal of Medical Education and Curricular Development. 2020;7.
158. Maulik PK, Tewari A, Devarapalli S, Kallakuri S, Patel A. The systematic medical appraisal, referral and treatment (smart) mental health project: Development and testing of electronic decision support system and formative research to understand perceptions about mental health in rural India. PLoS ONE. 2016;11(10).
159. Praharso NF, Pols H, Tiliopoulos N. Mental health literacy of Indonesian health practitioners and implications for mental health system development. Asian Journal of Psychiatry. 2020;54.
160. Subba P, Luitel NP, Kohrt BA, Jordans MJD. Improving detection of mental health problems in community settings in Nepal: Development and pilot testing of the community informant detection tool. Conflict and Health. 2017;11(1).
161. Jordans MJD, Luitel NP, Lund C, Kohrt BA. Evaluation of proactive community case detection to increase help seeking for mental health care: A pragmatic randomized controlled trial. Psychiatric Services. 2020;71(8):810-5.

162. Mills C, Hilberg E. 'Built for expansion': the 'social life' of the WHO's mental health GAP Intervention Guide. Sociology of health & illness. 2019;41(Supplement 1):162-75.
